# Genome to Phenome: Improving Animal Health, Production, and Well-Being – A New USDA Blueprint for Animal Genome Research 2018–2027

**DOI:** 10.3389/fgene.2019.00327

**Published:** 2019-05-16

**Authors:** Caird Rexroad, Jeffrey Vallet, Lakshmi Kumar Matukumalli, James Reecy, Derek Bickhart, Harvey Blackburn, Mark Boggess, Hans Cheng, Archie Clutter, Noelle Cockett, Catherine Ernst, Janet E. Fulton, John Liu, Joan Lunney, Holly Neibergs, Catherine Purcell, Timothy P. L. Smith, Tad Sonstegard, Jerry Taylor, Bhanu Telugu, Alison Van Eenennaam, Curtis P. Van Tassell, Kevin Wells

**Affiliations:** ^1^Office of National Programs, Agricultural Research Service, United States Department of Agriculture, Beltsville, MD, United States; ^2^National Institute of Food and Agriculture, United States Department of Agriculture, Washington, DC, United States; ^3^Department of Animal Science, Iowa State University, Ames, IA, United States; ^4^Dairy Forage Research Center, Agricultural Research Service, United States Department of Agriculture, Madison, WI, United States; ^5^National Animal Germplasm Program, Agricultural Research Service, United States Department of Agriculture, Fort Collins, CO, United States; ^6^Meat Animal Research Center, Agricultural Research Service, United States Department of Agriculture, Clay Center, NE, United States; ^7^Avian Disease and Oncology Laboratory, Agricultural Research Service, United States Department of Agriculture, East Lansing, MI, United States; ^8^Agricultural Research Division, University of Nebraska-Lincoln, Lincoln, NE, United States; ^9^President’s Office, Utah State University, Logan, UT, United States; ^10^Department of Animal Science, Michigan State University, East Lansing, MI, United States; ^11^Hy-Line International, West Des Moines, IA, United States; ^12^Department of Biology, College of Arts and Sciences, Syracuse University, Syracuse, NY, United States; ^13^Animal Parasitic Diseases Laboratory, Agricultural Research Service, United States Department of Agriculture, Beltsville, MD, United States; ^14^Department of Animal Sciences, Washington State University, Pullman, WA, United States; ^15^Department of Commerce, National Oceanic and Atmospheric Administration, La Jolla, CA, United States; ^16^Acceligen, A Recombinetics Company, St. Paul, MN, United States; ^17^Division of Animal Science, University of Missouri, Columbia, MO, United States; ^18^Department of Animal and Avian Sciences, University of Maryland, College Park, MD, United States; ^19^Department of Animal Science, University of California, Davis, Davis, CA, United States; ^20^Animal Genomics and Improvement Laboratory, Agricultural Research Service, United States Department of Agriculture, Beltsville, MD, United States

**Keywords:** animal, genomics, health, production, biotechnology, discovery, infrastructure, phenotype

## Abstract

In 2008, a consortium led by the Agricultural Research Service (ARS) and the National Institute for Food and Agriculture (NIFA) published the “Blueprint for USDA Efforts in Agricultural Animal Genomics 2008–2017,” which served as a guiding document for research and funding in animal genomics. In the decade that followed, many of the goals set forth in the blueprint were accomplished. However, several other goals require further research. In addition, new topics not covered in the original blueprint, which are the result of emerging technologies, require exploration. To develop a new, updated blueprint, ARS and NIFA, along with scientists in the animal genomics field, convened a workshop titled “Genome to Phenome: A USDA Blueprint for Improving Animal Production” in November 2017, and these discussions were used to develop new goals for the next decade. Like the previous blueprint, these goals are grouped into the broad categories “Science to Practice,” “Discovery Science,” and “Infrastructure.” New goals for characterizing the microbiome, enhancing the use of gene editing and other biotechnologies, and preserving genetic diversity are included in the new blueprint, along with updated goals within many genome research topics described in the previous blueprint. The updated blueprint that follows describes the vision, current state of the art, the research needed to advance the field, expected deliverables, and partnerships needed for each animal genomics research topic. Accomplishment of the goals described in the blueprint will significantly increase the ability to meet the demands for animal products by an increasing world population within the next decade.

## Introduction

U.S. agriculture must dramatically adapt many of its management practices and uses of natural resources if the Nation is to sustainably meet the food and fiber demands of current and future generations. USDA plays a vital role in facilitating scientific discoveries and technological innovation to ensure the availability of a safe, nutritious, and abundant food supply.

Agricultural animals have played a critical role in meeting human nutritional requirements for food and fiber. They currently provide 18% of the total calories and 39% of protein consumption (Food and Agriculture Organization of the United Nations [FAO], 2018). In addition to food, animal byproducts have many uses in pharmaceutical, cosmetic, household, and industrial products (Economic Research Service and USDA, 2011).

According to the FAO, the global population will approach 10 billion people by the year 2050 while the economic status of people in developing countries will continue to improve. As a result, there will be a profound increase in demand for animal products. Increasing animal production will require gaining a deeper understanding of animal biology through genomics and associated sciences to enable U.S. livestock, poultry, and aquaculture producers to maintain global competitiveness and adapt to changing climates and the need to reduce greenhouse gas emissions. At the same time, farmers will need to combat diseases in the face of increased antimicrobial resistance and pressure from consumers and regulators to minimize the use of antibiotics. Finally, animal welfare will be improved through new production systems and management practices.

*Animal production is a critical component of the U.S. economy*, with more than 1 million farms producing $182 billion in products in 2011 (National Agricultural Statistics Service [NASS], 2012) while employing more than 2.3 million people and representing 63.7% of farm income. In 2014, animal agriculture yielded $440.7 billion in economic output, with $76.7 billion in earnings, $19.6 billion in income taxes, and another $7.4 billion in property taxes (United Soybean Board, 2015). Countries such as Brazil^1^, India^2^, and China^3^ are increasing their investments in agricultural research to enhance their ability to contribute to world agricultural markets. To remain competitive, the United States must increase its investments in creating new strategies for animal production that meet the demands and values of consumers, and the increasing demands of the world population. The United States has a strong heritage of innovation in animal agriculture, and new technologies must be developed that increase efficiencies of production systems. These must include innovations that target animal health, nutrition, reproduction, and welfare such that the availability of a high-quality, safe, healthful, and affordable food supply is guaranteed.

The term “genome to phenome” describes the connection and causation between the genetic makeup of an animal (genome) and the totality of all phenotypes, or the observable physical or physiological traits or characteristics (phenome). Improving animal productivity will require a better understanding of the structure and function of animal genomes and how they interact with non-genetic components of production systems (e.g., nutrition, environment) so that management practices can be optimized to improve performance. Until recently, much of animal genomics research focused on sequencing animal genomes, detecting and cataloging sequence variants from individual animals, and then using that genomic variation to select for predicted genetic differences in routinely measured traits. Initially, obtaining genotypic information was the primary challenge; however, over the last decade, researchers, producers, and industry partners were incredibly successful at collecting a significant amount of genotypic and phenotypic data from large numbers of animals. Further work will be needed to continue to reduce the costs and increase performance of these genotyping platforms, particularly for industries in which the economic value of an individual animal is low relative to genotyping costs. However, for many agriculturally relevant species, the current challenge is to predict an animal’s phenotype based on its genotype and environment. Gene editing technologies, which will allow the interrogation of existing and novel genetic variation, will facilitate the identification of causal genetic variation. Understanding these genomic effects is now limited by the phenotypes that are collected. To efficiently use selection and modification of the genome of animals to accurately alter its phenome, an in-depth understanding of genome biology must be developed to dramatically expand capacity to characterize and measure phenomes. Much of this new blueprint is directed toward this purpose.

In 2008, national program leaders from the USDA Agricultural Research Service (ARS) and the National Institute of Food and Agriculture (NIFA) led the development of a report titled “Blueprint for USDA Efforts in Agricultural Animal Genomics 2008–2017” (2008 Blueprint) that presented a vision for implementing genomics to meet the challenges of animal production. The goals of this blueprint included accelerating animal breeding with the aims of livestock types with higher growth rates, reduced feed intake, improved fertility, and enhanced resistance to diseases. During the period targeted by the blueprint, USDA invested more than $500 million of research funding for projects addressing this vision. Many of the goals outlined in that original document were met or far exceeded expectations. The economic returns on genomic technologies that have affected the dairy cattle genetics sector alone have more than repaid this entire investment.

The USDA provided leadership among Federal agencies toward advancing genomics of agricultural animals and partnered with other funding agencies, including National Institutes of Health (NIH), National Science Foundation (NSF), Department of Energy (DOE), and U.S. Agency for International Development (USAID). The USDA also established and strengthened international partnerships with many scientific and funding bodies, such as European Commission (US-EU Task Force on Biotechnology Research, 2011), BBSRC in the United Kingdom (USDA and BBSRC, 2015), Genome Canada, CSIRO in Australia, AgResearch in New Zealand, EMBRAPA in Brazil, and INRA in France. Several large consortia were assembled as part of these collaborations to fund and coordinate large genome sequencing projects that led to the first genome assemblies for domestic poultry and livestock (African Goat Improvement Network [AGIN] Partner Organizations, 2018; USDA, 2018). In addition, several large consortia were created in direct response to proposal requests that were based on recommendations from the 2008 Blueprint document.

A decade later, leaders from the animal genomics community (Supplementary Appendixes 1, 2) have revisited this vision to reflect on changes in available genomic tools and reagents, genomics and computing technologies, consumer values, and the global ecosystem for animal production. The 2008 Blueprint focused on 13 species that were of economic interest in the United States; however, the document was intended to be species agnostic, recognizing that demand for animal products will be satisfied by many species. Equally important, the current threshold for cost and complexity of the infrastructure required to conduct genomic analyses for “new” species is considerably less expensive and time consuming, and these costs continue to fall. In November 2017, USDA and Iowa State University hosted Federal and university scientists, funding agencies, and industry stakeholders at a workshop^4^ with the aim of developing a collective vision for the next decade of animal genomics research (Supplementary Appendixes 3, 4).

The workshop included priorities communicated by the animal agriculture industries, perspectives of Federal and international funding agencies, and presentations from leaders in various fields of genome biology. Participants discussed a vision for how the next decade of genome research will be used to improve animal production, and the steps necessary to make those improvements a reality. The effort built on the successes of the previous report by identifying research priorities within the framework of the previous blueprint: (1) science to practice, (2) discovery science, and (3) infrastructure, while contributing to the following four overarching goals for animal production:

### Goal 1: Providing Nutritious Food for a Growing Human Population

Feed the growing human population, encompassing global food security, improving rural economies and development, increasing productivity of agricultural enterprise and exports of agricultural products, and reducing trade deficits.

### Goal 2: Improving Sustainability of Animal Agriculture

Improve environmental sustainability (reduce land and water usage, balance the use of antibiotics for animal health, and reduce greenhouse gas emissions), economic sustainability (consumer affordability and farmer profitability), and preserve germplasm and genetic diversity.

### Goal 3: Increasing Animal Fitness and Improving Animal Welfare

Improve animal fitness through adaptation to local and regional conditions (e.g., altitude), biotic and abiotic stresses such as climate change, diseases and pests, and optimizing the microbiome.

### Goal 4: Meeting Consumer Needs and Choices

Enable consumer choices such as cultural or traditional foods, healthy choices (lean and tender meat products), nutritional enhancements, and food raised through desired farming practices (i.e., organic, no antibiotics ever).

In July 2018 the National Academies of Sciences, Engineering and Medicine published a consensus study report titled “Science Breakthroughs to Advance Food and Agricultural Research by 2030^5^,” which was drafted by a committee of 13 experts charged with providing a broad new vision for food and agricultural research by outlining the most promising scientific breakthroughs to be anticipated over the next decade. The report included a vision for animal agriculture, specifically documenting the likely demand for an almost twofold increase in the availability of global animal protein through (1) a tenfold increase in the rate of genetic improvement and (2) the development of precision livestock production systems. The most recent advances in genomic selection, which are in various stages of implementation across animal species, have demonstrated up to a twofold increase. Therefore, another fivefold increase must come from a combination of biotechnology, advanced reproductive technologies, precision breeding strategies that better account for genetic by environment interactions, or some other advance not yet identified. The authors of the report made the following recommendations:

(1) Enable better disease detection and management using a data-driven approach through the development and use of sensing technologies and predictive algorithms.
(2) Accelerate genetic improvement in sustainability traits (such as fertility, improved feed efficiency, welfare, and disease resistance) in livestock, poultry, and aquaculture populations using big genotypic and sequence data sets linked to field phenotypes and combined with genomics, advanced reproductive technologies, and precision breeding techniques.
(3) Determine objective measures of sustainability and animal welfare, how those can be incorporated into precision livestock systems, and how the social sciences can inform and translate these scientific findings to promote consumer understanding of trade-offs and enable them to make informed purchasing decisions.

The authors of the report also highlighted the need for convergent approaches toward research, stating that “*The urgent progress needed today to address the most challenging problems requires leveraging capabilities across the scientific and technological enterprise in a convergent research approach*” and “*This means that merging diverse expertise areas stimulates innovation in both basic science discoveries and translational applications. Food and agricultural research needs to be broadened to harness advances in data science, materials science, and information technology*.” The vision for animal genomics in this 2018 Blueprint provides an additional layer of resolution that aligns with these research priorities for animal genetics, disease, nutrition, biotechnology, sustainability and welfare; and outlines the need to enhance workforce development and improve critical data and informatic infrastructures. The scientists of the animal genome community are well positioned to contribute to convergent research approaches that aim to meet the broad agricultural challenges facing the planet over the next decade and beyond.

## Science to Practice

### Genomic Selection in U.S. Animal Agriculture: Commercial Implementation of Genomic Technology

A primary goal of research in animal genomics is to use genomic information to improve the response of animals to selection. The best example of implementation of a new genomic technology in the last decade comes from the U.S. dairy cattle industry. The success of this effort depended on a collaborative network of scientists from ARS, land-grant universities, genetics companies, breed associations, and biotechnology companies. At the core of this network were important public/private partnerships that ranged from newly established relationships to long-standing partners. The common goal among all partners was to develop, deploy, and commercialize a genotyping assay that would dramatically improve selection accuracy in young animals, thereby allowing dramatic reductions in generation interval and the potential for selection on new traits such as feed efficiency.

**FIGURE 1:**
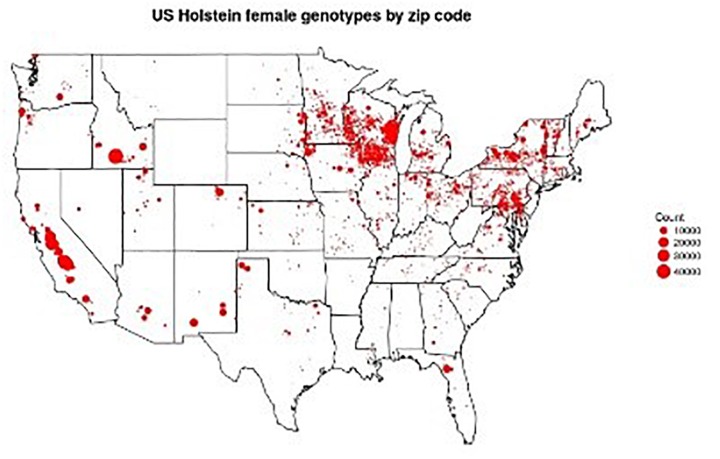
Locations of 1,136,252 genotyped Holsteins. Figure provided by Troy Rowan using zip code data contributed by George Wiggans.

**FIGURE 2:**
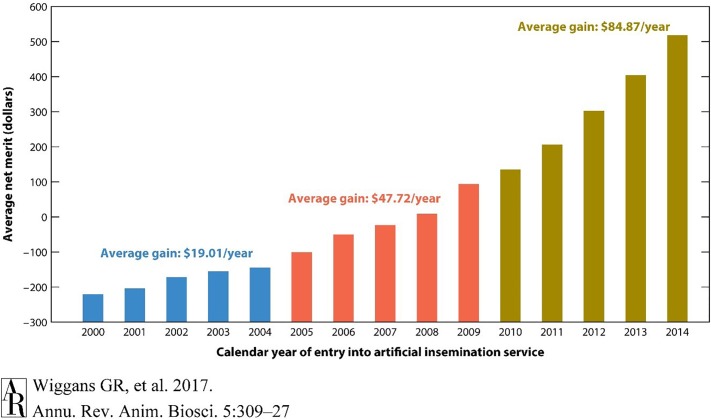
Average genetic value for net merit of artificial insemination bulls by year of entry into artificial insemination. The acceleration of genetic gain for net merit by incorporating genomic selection is illustrated by comparing rates of genetic gain across three time periods. Net merit is an index of traits designed to optimize productivity and profitability of daughters in a dairy herd. Genomic information from high-density genotyping was introduced in 2009. Reproduced from Wiggans and Cole (2017) which is not subject to copyright protection.

The dairy industry has led the agri-genomic revolution with the implementation of genome-enabled genetic predictions. In the United States, more than 2 million dairy cattle (mostly females) from 5 breeds have incorporated genome information into the national dairy genetic evaluation (Figure 1). The impact on genetic improvement has been profound. For example, lifetime net merit (NM$), an industry recognized index of many individual traits that predicts total economic value in dairy animals, approximately doubled in terms of profitability over the last 10 years (Figure 2). For traits with low heritabilities, the effect on genetic gain has been even more dramatic because the accuracy of predicted genetic values increases proportionally more than for more highly heritable traits. To take advantage of this phenomenon, genetic and genomic predictions recently became available for six health traits for Holsteins. These traits all have high economic values and low heritabilities, which makes them ideal traits in leveraging genomics.

**FIGURE 3:**
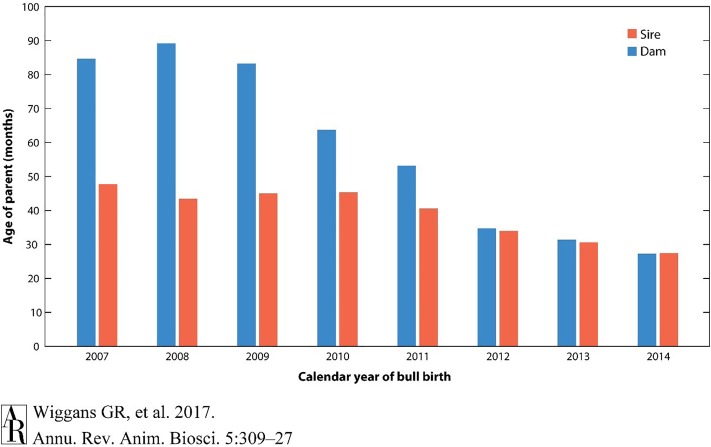
Reduction in the age of parents for selection in dairy cattle using genomic analysis. The generation interval has reduced to nearly one-third of that required without genomic selection. Reprinted from Wiggans and Cole (2017) which is not subject to copyright protection.

A large part of this dramatic change in genetic gain per year has been the striking decrease in generation intervals because of the ability to make selection decisions using DNA genotypes on very young animals without waiting until daughters are milked. The reduction in age of parents when a calf is born has changed most dramatically for bull calves, in which the age of sires has been reduced by half (Figure 3).

These results clearly show the acceleration of genetic gain rates that have been realized in dairy cattle. The total of USDA investment in dairy cattle genomics research over the last decade was approximately $100 million. Simple calculations made from Figure 2 suggest that the value of the addition of genomic selection to dairy cattle is worth approximately $50 per cow year. If this was implemented across the entire US dairy herd of 9 million cows, the calculated return would be $450 million dollars per year. Selection was implemented in 2009, 450 million multiplied by 9 years would provide a return on investment of $4 billion, which continues to accumulate. These estimates capture only a fraction of the economic impact that genomic selection has had on the dairy industry. For instance, before 2008, genotyping was primarily a research enterprise; genomic selection has now created an economy around the genotyping of commercial animals. Animal welfare has also been improved, as selection indices can now address common health issues including displaced abomasum, hypocalcemia (milk fever), ketosis, mastitis, metritis, and retained placenta^6^. Other aspects such as the export income from highly valued commercial germplasm from U.S. animals are also not quantified.

Over the last decade, genome-enabled technologies became integral components of commercial animal breeding for many species as advances in DNA sequencing and genotyping dramatically increased the ability to obtain genome information. Examples follow.

✓ The Angus breed in the beef industry has followed closely behind the dairy industry in using genome information from more than 500,000 commercial animals.
✓ Swine breeding companies actively use genome information to accelerate genetic improvement by about 30%, although details are confidential. Importantly, the commercial sector has participated in public-private partnerships to explore the use of genomics for difficult-to-measure traits. One example is efforts to develop genomic markers to mitigate the effects of Porcine Reproductive and Respiratory Syndrome.
✓ Poultry breeding companies are using genome information to accelerate genetic improvement in broilers and layers, although details of this are confidential. International research consortia are using genomics to identify chickens that are more resilient to the negative effects of heat stress (Monson and Angelica, 2018).
✓ Genome selection has been implemented in the commercial breeding of rainbow trout and Atlantic salmon; proof of concept has been demonstrated for catfish.

### Implementing Genome Science Into Animal Production

The information and infrastructure outlined in the following paragraphs will dramatically improve the ability to apply genome-enabled technologies in animal production. These efforts will be a continuation of those described in the previous animal genome blueprint. A brief description of successes and remaining gaps are discussed below.

The first goal described in the 2008 Blueprint was to establish *whole genome-enabled animal selection resulting in a significant reduction in selection cost and generation interval*. This goal has been successfully implemented in dairy cattle. To a lesser extent genomic selection has also been applied in beef cattle, swine, poultry, and fish. The success and rapid adoption of this selection by the dairy industry was influenced by the fact that only a single selected population exists within the dairy industry; dairy bulls are highly valuable because the semen of a single bull can be extensively used via artificial insemination (AI), and genomic information can be used in place of phenotyping, which could be measured in females only after long generation intervals. Nevertheless, new phenotypes continue to be collected within the dairy industry, and this is vital to the long-term sustainability of genomic selection. The beef cattle industry uses far less AI breeding, so individual bulls are less valuable; numerous breeds and populations with varying amounts of phenotypic record collection are used. The swine industry uses AI extensively, but many more sperm are required for adequate fertility, so individual boars are less valuable. Similar to beef cattle, numerous swine breeding companies exist within the United States, with separate and genetically distinct selected male and female lines. Poultry sires are even less valuable, making the cost of genotyping individual sires harder to justify economically, and the use of transported semen and artificial insemination is currently not viable. However, the use of genetically selected progeny for production is routine. Further progress in these species will require less expensive genotyping and the elucidation of DNA polymorphisms that change gene function and therefore traits of interest. Furthermore, continued reductions in the generation interval, enabled by genomic selection, will have dramatic effects on genetic advancement of animal populations.

The second goal in the 2008 Blueprint was *prediction of genetic merit from genome-based data combined with phenotypes*. This goal mostly has been achieved. However, current techniques assume that genomic effects are additive, and techniques to incorporate effects of heterosis are not yet available (see below). It remains difficult to know which DNA changes result in changes in phenotype, especially those changes that affect transcription. The collection of useful phenotypes is now the main factor limiting future progress. This goal requires the collection of trait phenotypes that are often complex and difficult to measure, and therefore expensive to obtain. Automated methods are needed to collect phenotypic measures in a cost-effective manner. A good example of this challenge is the combining of genomic information with feed efficiency measures into a unified data source. Automated methods of collecting feed efficiency are available, but they are expensive and somewhat challenging to use, and feed efficiency measures are therefore not widely available in many species. Better methods, or developing inexpensive correlated measures as indicators of efficiency, are needed to fully utilize genomic selection.

The third goal was the *integration of genomic data into large scale genetic evaluation programs and the use of genomic information to design precision mating systems*. The goal here was to begin to optimize heterosis (hybrid vigor) and epigenetic factors. This goal has not yet been achieved, but heterosis and epigenetic factors are beginning to be explored in earnest. Heterosis effects will be extremely important in swine and poultry industries where terminal sires and maternal line females are used specifically to take advantage of heterosis effects in offspring meant for production. Heterosis also affects commercial beef production, where nearly all slaughter animals are crossbred. It is possible that in animals in which heterosis is appropriately used, the resulting improvement in production may be as great as that from genomic selection based on additive inheritance.

The fourth goal was the development of *precision management systems to optimize animal production*. This is similar in concept to precision therapeutic treatments in humans, which is beginning to be used in the treatment of certain cancers by genotyping the cancer cells and adjusting the treatments accordingly (Leão and Ahmed, 2019). An example of this approach in livestock has already been implemented in dairy cattle, where separate genetic merits for cheese making (Francesco Tiezzi, 2018) and pasture-managed dairy cattle (Newton and Hayes, 2018) have been developed. Aside from this example, precision management based on genotype is still in its infancy.

The final goal from the 2008 Blueprint was the development of *genomic capabilities that enable parentage and identity verification (traceability)*. This goal has largely been achieved because several technologies and strategies are available that can be used to genotype a few dozen to a few thousand genetic markers to permit parentage or population assignments. However, genotyping is still too expensive to implement widely because the value of individuals, and the parentage information that results, do not justify the cost.

**FIGURE 4:**
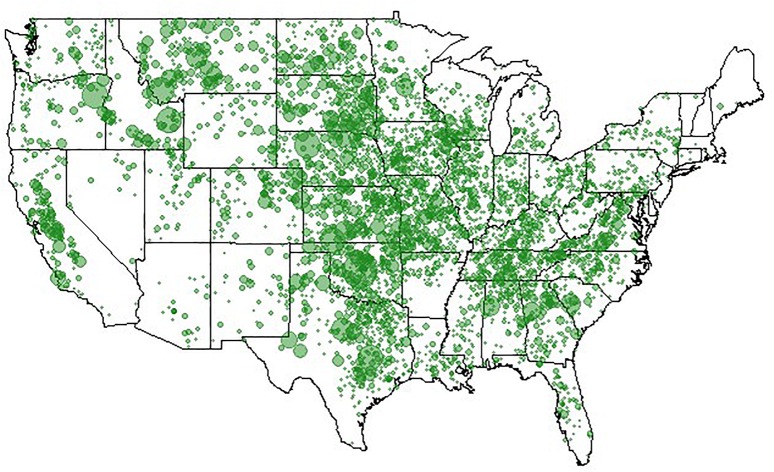
Locations of 521,645 genotyped animals. Figure provided by of Dan Moser, American Angus Association.

**FIGURE 5:**
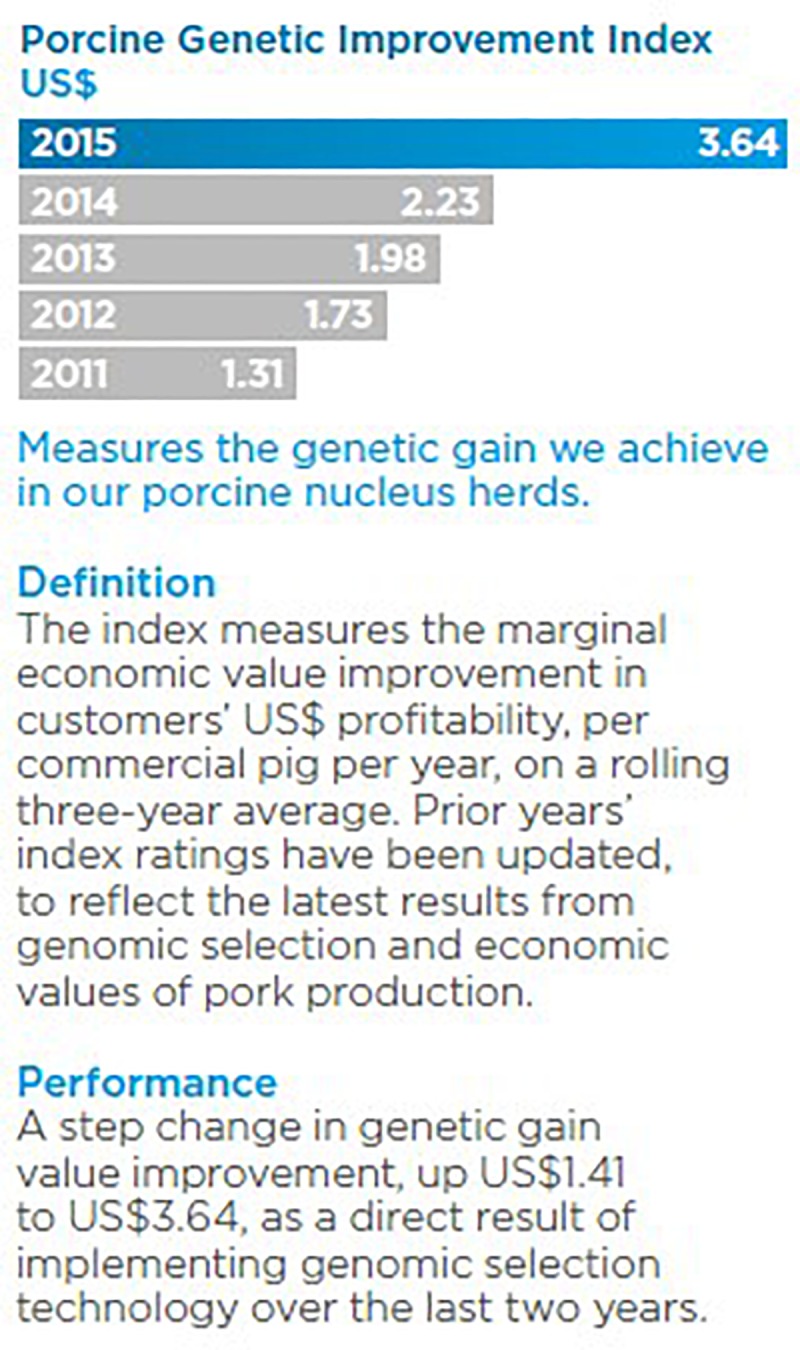
The Porcine Genetic Improvement in US Dollars index shows the genetic gain achieved in the nucleus hers of the Pig Improvement Company (PIC). The index shows the genetic gain achieved in the porcine nucleus herds of PIC, the world’s largest porcine breeding company. It measures the marginal economic value of improvement in customer profitability. A greater increase (>35% long term) in the rate of change in genetic gain as a direct result of implementing genomic selection has been achieved. Reproduced as a courtesy from Ernst Van Orsouw from the Genus Annual Report 2015 (p. 16, https://www.genusplc.com/investors/results-reports-and-presentations/).

**FIGURE 6:**
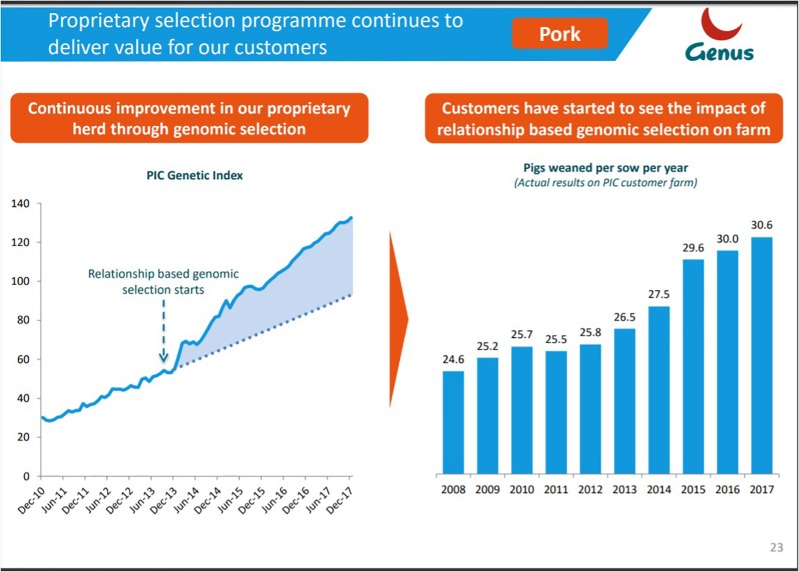
Genetic improvement in a nucleus herd translates into visible gains on commercial farms. Reproduced as a courtesy from Ernst Van Orsouw from the Genus 2018 investor presentation (https://www.genusplc.com/media/1460/genus-interim-results-presentation-28feb2018.pdf).

**FIGURE 7:**
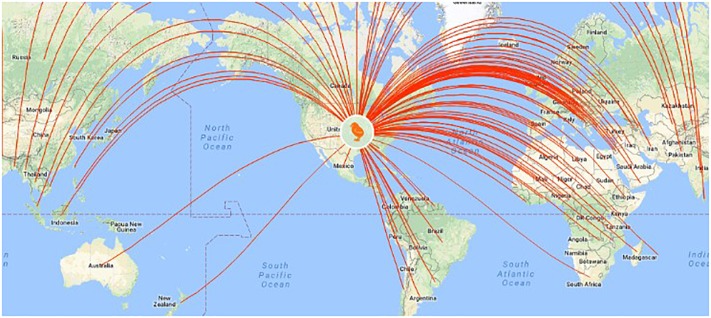
U.S. poultry companies export genome-selected poultry breeding stock to 110 countries (http://www.hyline.com/UserDocs/Pages/INNO_ISSUE_15_ENG.pdf).

**FIGURE 8:**
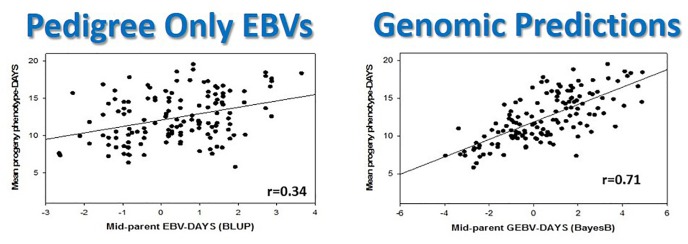
Genomic selection in rainbow trout doubles selection accuracy in a single generation compared with traditional pedigree-based predictions. This figure is reproduced and modified from Additional file 2 of Figure S1 in Vallejo and Leeds (2017).

Clearly, animal genomics has come a long way in the quest for efficient genome-enabled selection, as evidenced by nationwide genotyping of commercial cattle (Figure 4), realizing increased genetic gains through genomic selection of pigs (Figure 5, 6), export of genome selected poultry (Figure 7) and increased selection accuracy in rainbow trout (Figure 8).

### Optimizing Animal Production Through Precision Breeding and Management

#### Vision

Genome-based analyses that increase knowledge of the genetic basis of production traits will lead to the development of new technologies that optimize animal breeding strategies and inform management decisions to realize the maximum production potential of animals across environments.

#### Current State of the Art

The primary use of genome-enabled technologies is the identification of large numbers of single nucleotide polymorphism markers that represent genetic diversity at the molecular level. These are associated with economically important traits. High-throughput platforms for genotyping large numbers of these markers in a single assay has led to their widespread use in research that seeks to determine the genetic basis of traits and improve predictions of performance in offspring.

Creating successful genome-enabled applications for commercial breeding industries was facilitated by many factors:

(1) Availability of species-specific genome tools and reagents;
(2) Advances in genome technologies that increased the ability to collect data at lower costs;
(3) Access to contemporary and historical DNA samples that correspond to performance information from large numbers of commercially relevant animals;
(4) Public-private partnerships between researchers and industries, breed associations and/or large companies with centralized breeding programs; and
(5) Genetic evaluation models for which independent genetic and environment components are added to determine phenotype.

Progress in commercial breeding attributed to genomics has been primarily achieved through four practices: (1) identifying and selecting for one or a few alleles having significant effects on phenotype; (2) using large numbers of genetic marker combinations to more correctly define relatedness between animals in a population and therefore increase selection accuracies; (3) identifying and eliminating deleterious recessive alleles that reside in populations because they lay dormant when inherited from a single parent but are fully expressed when inherited from both; and (4) identifying animals with higher genome estimated breeding values (e.g., sex- or age-limited traits) earlier; for example, egg layer chickens for which young males can be genomically selected for traits expressed only in females and that can be measured only in older females (egg production over extended periods). The technologies that enable these advances in breeding have just begun to unlock the potential of genomic techniques to improve animal production.

#### Advancing the State of the Art

Improving the use of genome information in commercial animal production will require the efforts described here.

##### Continue the development of species-specific genome tools and resources

Access to species-specific genome information, including well-annotated genome reference assemblies, characterization of genetic diversity within and across breeds, and developing cost-effective methods to carry out genome analyses, are critical if we are to make improvements over contemporary strategies. Cost-effectiveness is partially dependent on data sharing and reproducibility.

##### Expand traceability applications

Genome-enabled parentage and traceability are used to address questions associated with species management and product quality. Traceability can also address food safety concerns and tie in with “food to fork” initiatives but this has not really been addressed in US livestock production the same way it has in other countries^7^. For another example, cultured and wild aquaculture species are often at risk for mixing. As offshore aquaculture develops [for example, in the Gulf of Mexico where 18 species are being considered for farming (Florida Atlantic University, 2017)], genome analyses are needed to verify broodfish region of origin. This would minimize the risks to wild fish if cultured fish escape and provide traceability tools if a sizable escapement episode occurs^8^. Genomic-scale genotyping of broodstock populations is attractive for some aquaculture species in which breeding candidates are limited to variant selection from wild-caught or F1 brood individuals in an effort to mitigate escapement concerns.

##### Expand current genome-enabled technologies to additional species

Genome-enabled technologies have been developed for a few species with high economic value and research investments. However, it is possible to make further improvements in many of these species and employing these technologies in additional species and industries has great potential to improve animal production. For instance, although the United States contributed to the development of genome tools for sheep, the use of genome information has been limited compared with New Zealand, for example, which has large sheep populations and where genomic information is used extensively (van der Werf et al., 2014; McEwan, 2016). Similarly, the use of genome information to improve goat genetics is not currently practiced in the United States, but it is used in Europe to improve dairy goat populations (Carillier et al., 2013; Antonio Molina, 2018). Since genome-enabled technologies have been applied where there is a clear economic gain, then there is rationale to be made for similar investments in other species. Consumers and producers are also poised to benefit by applying genome-enabled technologies to aquaculture species whose domestic production was only recently initiated, such as oysters and the California Yellowtail marine fish. To implement improvements in genome-enabled selection in more animal species, scientists must do the following:

(1) Use genomic data to determine relatedness among individuals to calculate genetic merits or “GBLUP.”
(2) Use genomic marker data to implement genomic selection.
(3) Supplement genomic selection with the use of identified causative mutations.
(4) Supplement genotyping with genome sequence data to detect all common DNA variation in individuals.
(5) Expand data sets to include trait-relevant transcriptomic, proteomic, and metabolomic information.
(6) Develop and implement strategies that reduce impacts of inbreeding.

##### Collect new and more extensive phenotypes

Researchers must have access to large integrated data sets of easily searchable phenotypic, environmental, and genomic information. Ideally, comprehensive phenotypes representing a wide range of production, reproduction, fitness, metabolic, welfare, disease susceptibility, and immune responsiveness traits would be collected on every commercial animal to allow precision breeding (see below). Phenotype and trait ontologies exist in animal genomics, however their utilization must increase to maximize the value of data sets.

##### Identify causal alleles

Identifying and characterizing the alleles that directly affect the biochemical mechanisms that underlie differences in phenotypes and therefore economically important traits will significantly enhance efforts to optimize breeding strategies. Many important production traits are multigenic and their regulatory elements are largely undetermined, which creates a gap in translating genotype to phenotype that must be addressed.

##### Implement precision breeding

Except for dairy cattle and poultry (egg layers vs. broilers), genomic analyses do not account for the diverse products for which the animals will be used, or potentially different management systems. Contemporary genetic models treat genetics and environment as independent factors with additive effects. But the reality is much more complex, and models should reflect the complexity of biology by effectively incorporating non-additive effects such as genetic × environment interactions, epistasis, epigenetics, inbreeding depression, and heterosis. These models must also expand to include the effects of management practices and socioeconomic factors (e.g., greenhouse gas production, organic production methods). The following capacities are needed to implement precision breeding:

(1) Comprehensive collection of relevant environmental (including management), genotype, and phenotype information across a broad array of commercial settings in common formats and data standards along with metadata for community access and use.
(2) Comprehensive, inexpensive and reliable measures of DNA nucleotide methylation and chromatin state within tissues that are relevant to production phenotypes.
(3) Genome-wide methods for the incorporation of non-additive effects into genomic analysis.

##### Implement precision management

Increasing the volume of genomic and production data collected on individual animals across production environments will enhance the ability to select animals for desired performance traits. Knowledge of an animal’s genetic potential for a suite of traits across a set of production environments will allow the development of models and algorithms for the precise sorting of animals into production environments that will maximize productivity and profitability and will also enhance animal well-being. Furthermore, effective incorporation of environmental and genetic information will facilitate the prediction of individual phenotypes. Examples include:

(1) Predicted phenotypes for frame size, entry weight, growth rate, feed efficiency, and marbling that could be used to assign groups for finishing at the feedlot.
(2) Different genetic merits could be calculated that are more conducive to outdoor rather than confinement systems.
(3) Genotypes may allow the production of specialty food products (e.g., β-lactoglobulin and κ-casein genotypes and milk destined for cheese manufacturing).
(4) Genotypes that predict health and production at different altitudes, or at different thermal indexes.
(5) Feeding regimens and preventive health care programs could be designed to match an animal’s genotype, which would lead to increased production efficiency, targeted market endpoints, and new opportunities for niche market production systems.

##### Integrate genome-enabled selection and biotechnology

Combining genome-enabled breeding with biotechnological applications such as gene editing of causal alleles will further optimize genetic improvement. Furthermore, the use of gene editing combined with advanced reproductive technologies (e.g., *in vitro* fertilization; cloning) have the potential to dramatically reduce the generation interval from years to weeks or days.

#### Resources Required

Advancing animal production through precision breeding and management will require a data savvy workforce trained in quantitative and molecular genetics, centralized animal populations to serve as resources for phenotyping, new technologies for phenotype collection, long-term data infrastructure, computational resources, high-quality and cost-effective genome sequencing and genotyping platforms, development of new analysis and prediction algorithms, and enhanced reproductive technologies.

#### Expected Impacts and Deliverables

The next decade will build upon the success of the previous decade by expanding genome-enabled selection to additional species, establishing new traits through genetic improvement for all species, and establishing more complex models for predicting phenotype from genotype and environmental variables. This includes strategies to harness the effects of heterosis and epigenetic mechanisms, genomic selection that fits specific environments (including those created by management), and designer pre/probiotics or metabolites that work effectively with different selected genomes. These and other technologies are expected to enhance productivity, sustainability, and profitability while improving animal well-being, health, and overall fitness.

#### Partnerships

Public-private partnerships will ensure the usefulness and application of research results for commercial breeding and management practices and final food processing. Partnerships between researchers, breeding and animal health companies, and breed associations will be critical. Collaboration with the international animal genomics community will ensure the adoption of state-of-the-art technologies, while partnerships with breeding and technology companies will be critical for developing animals for optimal purposes through precision breeding and management.

## Discovery Science

Developing new technologies that improve aspects of animal production requires gaining a thorough understanding of animal biology. Over the last decade, US scientists and their international colleagues and collaborators have used genomic approaches to dramatically increase the biological knowledge base for agricultural animal species. Since 2008, 7,558 peer-reviewed publications indexed in PubMed reported work related to genomics of the 13 species highlighted in the 2008 Blueprint, almost doubling the output of the previous decade and constituting almost 44% of these types of publications since 1949 (Table 1 and Supplementary Appendix 5a).

**Table 1:** Publications associated with genomics of animal production.

Time Frame	No. of Pubs
2008–2017	7,558
1998–2007	3,730
Total since 1949	17,203

Greater knowledge of genome biology is not only reflected in the number of publications, but also in the amount of available genome information. During this time, new DNA sequencing technologies changed the capacity, efficiency, and affordability of obtaining genome information, as reflected in Supplementary Appendix 5b. As a direct result, there have been dramatic increases in the single nucleotide polymorphism database (dbSNP, Supplementary Appendix 5c) and short-read archive (SRA, Supplementary Appendix 5d) for the 13 species highlighted in the 2008 Blueprint, as well as the addition of significant information on genetic markers associated with traits of economic importance (QTLdb^9^, Supplementary Appendix 5e). Although NCBI dbSNP no longer contains information for livestock species, it is available at Ensembl^10^.

In the 2008 Blueprint, the first goal for discovery science was to *identify genes and gene products that regulate important traits in agricultural animals.* There have been numerous successes for this goal. Most of these achievements were discoveries of very large single-gene effects [e.g., myostatin (McPherron and Lee, 1997) and callipyge (Cockett et al., 2005) and mutations that altered muscle development, a DGAT (Grisart and Coppieters, 2002) genetic variant that affected milk fat]. There have been few successes in finding mutations associated with smaller differences relative to the overall trait variation. However, the accelerated growth in availability of genome sequences for animals has provided increasing numbers of candidate variants. Using bioinformatics tools that can predict the effect of simple changes in the genome, variants have been categorized based on severity of change in the form and function of the proteins and/or RNAs coded by their respective genes (Weller and Bickhart, 2018). The effects these sequences may have on changes in performance traits are just beginning to be explored.

The second goal was to *understand mechanisms that regulate agriculturally relevant genes in a systems biology framework.* The availability of high-quality reference genomes has enabled the identification and characterization of many regulatory elements in animal genomes. Improvements in genome sequencing technology has resulted in more accurate determination of gene transcript information. Current technologies even allow the direct sequencing of RNAs from a single cell. These technologies represent a quantum leap in the ability to measure gene expression. However, challenges remain in understanding the regulation and expression of genes across developmental stages and different tissues. Modeling and understanding the complexities of the control of gene expression is still a work in progress. The questions to be answered are identical to those asked by the Human Encode (encyclopedia of DNA elements) Project; however, the animal genomics community is much smaller and has many fewer resources. The Encode project integrates experimental evidence from many different sources (e.g., methylation levels, histone marks) to identify motifs or variants that are related to gene regulation ^11^. In 2015, the Functional Annotation of Animal Genomes (FAANG) consortium was organized to provide the same information in animals that is currently available for humans. Results are beginning to become available from these efforts (Guiffra and Tuggle, 2018). This ability to *define the mechanisms through which specific genes and genetic variation influence phenotypes and phenotypic variation* was the third goal of the section on discovery science from the original 2008 Blueprint.

The fourth goal was to *understand the roles and interactions of host animal and microbial genomes and environmental influences for improving animal health, well-being, and production efficiency*. The first challenge to addressing this goal is to ensure complete characterization of the microbes represented in a specific environment. Historically, techniques based on variation in ribosomal genes of microbes have been used to catalog the microbes present (i.e., the metagenome). These techniques have been used successfully to characterize microbial populations in the rumen, respiratory system, digestive tract, and feces of livestock species, although at a low level of taxonomic resolution. Strong evidence exists that variation in these microbial populations affects performance and disease responses (Zeineldin and Barakat, 2017; Difford et al., 2018; Li and Hitch, 2019). Unfortunately, ribosomal RNA genes do not provide a complete picture of the different microbes in the microbial community, so alternative strategies are being explored. One effective strategy has been to focus on the enzymatic activity of the genes present in the combined population (Xing and Zhang, 2012; Ribeiro and Gruninger, 2016; Santosh Thapa, 2017). Another avenue of investigation is to sequence and assemble the genomes of all the microbes in the microbial community. These and even more creative strategies will be needed to fully understand and eventually manipulate these populations to optimize production.

The sections that follow describe the needs for discovery science that follows from the 2008 Blueprint. To make genomic selection more effective and enable management techniques that rely on how genes function in animal species, a detailed understanding of how individual genes and their alleles affect phenotypes will be required. An area in which genomic technologies could have a profound revolutionary effect is in animal health and welfare, both in selection of animals to be more tolerant or resistant to diseases, and in genome-enabled strategies that might be used to improve animal health and treatment of sick animals. Greater numbers of less expensive and more detailed phenotypes are needed to advance genomic selection. Finally, the genome of an individual food-producing animal is not the only genome that influences its phenotype. The phenotype of all food-producing animals is also influenced by its microbiome, which consists of the parasitic, commensal, and symbiotic organisms that exist within one or more organ systems.

### Understanding Genome Biology to Accelerate Genetic Improvement of Economically Important Traits

#### Vision

Identification and characterization of the genes and biochemical mechanisms that underlie variation in traits associated with animal health and production efficiency will accelerate genetic improvement through the incorporation of molecular phenotypes.

#### Current State of the Art

Most of our knowledge of genome biology comes from discoveries in model organisms that are only somewhat related to agricultural animals. However, given the ease of comparing sequence information across species, there is limited confidence for predicting gene function from humans and model organisms such as mice, rats, and zebrafish to agriculturally important species; these predictions need to be confirmed. There is very limited direct experimental evidence of function for the majority of genes in agricultural animals. Most genes have unknown functions or have functions inferred from observations in other species, and many of those predicted functions may not be relevant to agriculture. Comparative information is also limited because agriculturally important traits are not typically studied in model species. This limits the ability to identify and validate causative DNA sequence variants potentially associated with economically important traits.

For many agricultural animals with advanced genome reference assemblies, most protein-coding genes are easily predicted and validated through transcriptomic and proteomic data, even when the function of the gene is unknown. However, differential RNA splicing contributes a great deal of variation to the proteins that can be coded by a single gene, and differential splicing in agricultural animals is poorly characterized. Non-protein-coding genes are difficult to predict and validate; therefore, they are also poorly characterized. Finally, very little is understood about regulatory sequences (promoters, enhancers, repressors, etc.), that are critical factors in regulating the genes underlying complex trait phenotypes.

In biomedicine the state of the art in genome biology includes the identification and analyses of orthologous phenotypes (phenologs) which reveal conserved gene networks that facilitate identification of candidate genes (Kriston and McGary, 2010), this approach is hindered in livestock as it relies on using standardized ontology structures.

#### Advancing the State of the Art

Discovery of gene functions that are directly associated with economically important traits in agricultural animals will facilitate the identification of biochemical and genetic markers that can be indexed in genetic improvement programs. This will require the tasks described below.

##### Catalog gene expression

Determining mechanisms that underlie phenotypic variation and regulation of gene expression across tissues and biological states is essential to incorporate molecular phenotypes into genetic improvement programs. Expression profiling of genes across diverse tissues, animal populations, and environmental conditions using next-generation sequencing approaches will identify novel genes and characterize spatial and temporal patterns of expression and differential splicing, as well as indicate functional associations with complex phenotypes. This includes analyses of gene expression that target specific performance characteristics and employ strategies to detect differences between commercial animals and their unselected ancestors. For example, the ancestral chicken that produced 6–12 eggs within the first year of life can be contrasted with the commercial egg-laying hen, which can be expected to produce more than 200 eggs by 52 weeks of age, and to continue to produce at a high rate for another 50 weeks or more.

##### Link genes to function

Despite the ability to predict protein sequences from DNA sequences, the functions of many proteins remain unknown. In some cases, even when the protein sequence predicts a possible enzymatic or other function, the actual ligands involved are not known. Phenotypes are the ultimate result of the molecular cascade that proceeds from gene (DNA) to transcript(s) (RNA) to protein(s) to metabolic substrate(s) and are linked to specific tissues, cell subsets, and fluids. Better transcriptomic, proteomic, and metabolomic technologies (including enhanced technologies and analysis methods) are needed to fully bridge the gap between DNA sequence and phenotype; to help identify the genes, proteins, and epigenetic modifications controlling various phenotypes; and to help identify the DNA changes responsible for different phenotypes.

##### Discover and exploit epigenetic factors

Determining functional elements of gene expression, including epigenetic modifications of the DNA and chromatin structure, will improve the accuracy of phenotype prediction. As previously described, the FAANG project aims to produce comprehensive maps of functional elements in the genomes of domesticated animal species that affect transcription. With results generated by the FAANG effort, bioinformatic scientists will be able to identify biologically significant DNA sequence variation that affects gene transcription and understand the complexity of gene function, including mapping transcriptomic factors that control gene and protein expression.

##### Standardize frameworks for functional genomics data

Establishing common assays, solid working standards, frameworks and infrastructure for data collection will enable integration of data sets among scientists and cross-species comparisons. Tools can then be developed for wider metadata analyses that leverage the collective expertise and resources of the animal genomics community.

##### Establish high-quality, functionally annotated genome reference sequences

The development of high-quality, gap-free reference genome sequences that encompass genetic diversity, highlight functional elements, and provide relevant annotation for agricultural animal species will enable interpretation of genome information within and across species.

#### Resources Required

Increased understanding of genome biology will require an agricultural workforce trained in molecular biology and infrastructure for collecting, storing, analyzing and sharing functional genomic data across the animal genomics community. The amount of stored information will be substantial, so a permanent place for these data to reside, along with well-developed software that allows easy access and analysis of these data by the research community, is essential.

#### Expected Impacts and Deliverables

Application of current and emerging genomic, proteomic, and metabolomic technologies from human and model species will improve annotation of agricultural animal genomes and identification of functional variants and will provide information that can be used to develop strategies that improve animal production. Expected outcomes and deliverables include:

✓ Functional annotation to help understand developmental stages from embryos to neonates and adults. Strategies will be developed that predict performance in diverse biological and physiological states throughout the productive lives of agricultural animals.
✓ Comparisons and correlations of results from cells, tissues, and whole animals to reveal regulatory networks that determine the suitability of using *in vitro* models for trait improvement instead of whole animals.
✓ Understanding the complexities of tissue and body fluids, and their proteomes and metabolomes, to help differentiate phenotypic variations.
✓ Identification of the importance of the three-dimensional structure of chromatin on genome function and its effects on animal performance.
✓ Completion of the first genome-wide maps for chromatin state for agricultural animals that include locations of functional elements.
✓ Characterization of the roles of microRNAs; other small non-coding RNAs, and other non-protein-coding genes on genome functions.
✓ Use of functional information within and across species to identify targets for genome modification and validate their effects on phenotypes.
✓ Discovery of the relationships between the regulation of gene expression, genetic variation, and trait expression to build networks and models that predict phenotype.

#### Partnerships

Successfully incorporating discoveries from genome biology into strategies that enhance animal production will require partnerships between the animal genomics community and data scientists, the biomedical community, evolutionary biologists, and animal health and breeding companies. This will ensure current and emerging technologies are effectively tailored for agricultural applications and novel products.

### Reducing the Effects of Animal Diseases

#### Vision

Multidisciplinary and coordinated research strategies that employ genomic techniques will define the biological determinants of disease and accelerate the development of disease-resistant or -resilient animals, effective vaccines, probiotics, and management practices.

#### Current State of the Art

Infectious disease is the unfavorable outcome of a pathogen infection of a susceptible host under certain environmental conditions. Diseases of livestock and aquaculture animals are estimated to cost U.S. agriculture more than $6 billion each year, and significantly affect industry profitability and trade opportunities. Beyond the economic costs, there are serious rising concerns about animal welfare and antimicrobial use that are compounded by pests and pathogens. Thus, a critical need exists to understand the fundamental biological determinants of disease, which can then be translated into the production of disease-resistant or -resilient animals, preventive therapeutics, and management practices that promote healthy animals and safe and affordable food products and minimize zoonotic diseases.

A fundamental issue for disease resistance traits is that resistance is measurable only in the presence of the disease-causing pathogen. In addition, for most agricultural animals, the genes and products of the innate and adaptive immune system are not fully known or functionally annotated. Many immune-related genes exist as multiple copies within an individual animal, and the number, sequence, and regulation of these similar genes are difficult to characterize. It is generally true that animal genomics researchers are in the early stages of being able to identify genetic variation that accounts for disease resistance or tolerance. The lack of methods to follow specific genes or to functionally measure outputs at the cellular or whole animal levels limits our ability to fill the knowledge gaps. Many diseases are complex, and their causative pathogens are unknown. The influence of a healthy microbiome on pathogen virulence is only now beginning to be understood. Vaccines for some pathogens are available, but few are 100% effective and there is fear that their widespread use will promote the evolution of more virulent pathogens.

Diagnostic laboratories exist in many parts of the country but reports of diseases associated with pathogens are coordinated only for regulated pathogens and diseases of major concern. For other diseases, there are unfortunately no repositories for sample storage, which hinders efforts to monitor and control disease and to study pathogen evolution and spread.

#### Advancing the State of the Art

A comprehensive understanding of the genetic basis for host susceptibility, resistance, and tolerance to pathogens is sorely needed to inform the development of strategies that lead to integrated approaches for reducing instances of disease and to ensure animal health and well-being. This will require the following actions:

##### Share information

Development and sharing of knowledge about precise phenotypes associated with disease, from initial infection to immune response and final pathogen clearance (or persistence) is needed; this includes diagnostic data to facilitate community-based integrated approaches to disease management.

##### Develop tools for phenotyping

Equipment and facilities that enhance and automate precision phenotyping of animals with disease must be developed. Critical phenotypes must be defined that focus on the identification of genes and polymorphisms, transcript isoforms, proteins, regulatory elements, and other genetic underpinnings of immunological and physiological responses to pathogens. This would include assays for all food-animal species to analyze cytokines and other immune biomarkers and cellular responses, identification of pathways, and networks associated with pathogen recognition and clearance. This will require verification of these responses from the single cell to systemic levels.

##### Examine host-pathogen-environment interactions

Knowledge of how vaccines, prebiotics and probiotics, vectors, and host microbiomes function and interact with the host will greatly aid efforts to develop sustainable strategies for disease control. Greater understanding of how abiotic factors influence disease susceptibility and tolerance is also required. Ultimately, there should be a comprehensive understanding of the general and species-specific adaptive and innate immune response to all agriculturally important pathogens. Large-scale screening for comprehensive host-pathogen interactions will aid efforts to determine the molecular basis for this complex interaction. National laboratories or large collaborative efforts should be assembled or enhanced that (1) maintain databases on pathogen frequencies for diseases by geography, (2) provide a national surveillance system with enhanced genomic assay tools, (3) provide repositories for known or suspected pathogens, (4) provide repositories for DNA and tissue samples from animals affected by disease, and (5) provide facilities for controlled challenge experiments.

##### Identify pathogens and diagnose diseases

Causative pathogens must be identified, especially when many may be present in the environment, and methods must be developed to ensure rapid, affordable, and accurate diagnostics that also distinguish between the immune response from pathogens and the immune response from vaccines. Reemerging and new diseases, including multifactorial diseases, need to be addressed due to changes in management, climate, and other abiotic factors. Host genomic and immunological knowledge will be integrated with outbreak tracking tools and complete annotated genomes and pathogen variance characterization to provide information associated with disease phenotypes for a comprehensive understanding of the roles of host, pathogen, vector (if applicable), and environment in health and disease. Note that only a very small proportion of the bacterial and viral species that exist on the planet have been identified and characterized, and that there are very likely many completely unidentified pathogenic species that remain to be discovered. Moreover, the complex interactions of a healthy microbiome in preventing pathogen replication and controlling disease and vaccine responses is only now being explored. Genomics (and in particular metagenomics) can help us begin to identify the microbial species that are actually present and affecting animal production systems.

Ultimately, multidisciplinary integration of information gleaned through genomics, computational biology, immunology, pathology, virology and bacteriology, and animal husbandry will be required if we are to have a better understanding of host-pathogen interactions.

#### Expected Impacts and Deliverables

This research will result in greatly reduced direct and indirect costs associated with animal disease, maintenance of a secure and safe food supply; improved animal welfare, production efficiency and resilience to environmental changes; and reductions in antimicrobial use and improved vaccines or other measures that can mitigate or prevent existing, new, and re-emerging infectious pathogens. Technologies will be developed that distinguish between vaccinated and infected animals and diagnostics that identify multiple pathogens within a sample.

#### Partnerships

Successful implementation will require the coordination of state, national, and international government agencies, especially those involved in animal health monitoring and diagnostics. Partnerships with producers and animal health companies with access to natural outbreak tissue and pathogen samples will be of high value.

### Applying Precision Agriculture Technologies to Animal Phenotyping

#### Vision

Development and deployment of state-of-the-art sensor technologies will enhance animal production through the creation of new ways to increase the accuracy of genomic predictions of superior phenotypic performance.

#### Current State of the Art

Agricultural animals are selected for favorable traits that can be reliably measured in large numbers of animals. Genotyping animals at numerous genetic variant densities continues to become easier and cheaper. Interestingly, this has resulted in phenotype collection becoming the rate-limiting step to genetic improvement. Most current phenotyping methods are labor intensive. Automation of phenotype collection is desperately needed, including data management plans that address formatting, sharing and access. Some automated phenotyping already exists (e.g., milk, fat, and protein content in dairy cattle), but the implementation of automated measures in most animal species is in its infancy. For fundamental advances to occur in low and moderately heritable traits, it will be imperative to accurately measure large numbers of complex phenotypes on a large number of animals for traits associated with fitness and economic value.

#### Advancing the State of the Art

Development of new sensors, technologies, and methodologies to automate the collection of phenotypes associated with disease resistance, animal well-being, reproduction, feed efficiency, and product quality in agricultural animals will enhance genetic improvement programs and inform management practices to optimize animal production systems. This will require the following:

##### New phenotypes

Development of methods for the measurement of existing and new phenotypes while investigating the basic biology underlying these traits is needed. Phenotypes to be collected on a routine basis could range from direct measurements of traits of individual animals (e.g., production, reproduction, health), the metagenome (numerous locations within the animal), the proteome/metabolome (numerous tissues), and functional genomic assays (e.g., transcriptome, methylation, histone acetylation, etc.).

##### Gene × environment interactions

Integration of geospatial and environmental data to account for more variation in phenotypic data will enhance analyses and allow for precision selection and management. For example, environmental variation may help to better account for pathogen transmission and disease parameters; these will enable improved identification of resistance/susceptibility to pathogens. Adapting animals to heat tolerance will be needed as climate change begins to alter the thermal characteristics of production environments. Animals may no longer be well adapted as heat and humidity profiles change.

##### High-throughput data collection

High throughput methods are needed that provide accurate measures that are relevant to economic traits and are not as labor-intensive to collect. This will require the development of new sensors and automated methods of data acquisition on the farm. An understanding of the relationships between high-throughput measures (e.g., host animal microbial communities, proteomes, and metabolomes; and environmental influences such as animal feed and vaccines) with indicators of animal health, well-being, and production efficiency, etc. is critically necessary. New analytical/statistical methods that are easily incorporated into high-throughput phenotyping methods used in commercial animal production (e.g., genomic selection, precision management, etc.) must be developed.

##### Data infrastructure

High throughput methods will generate a lot of data. To accommodate the data volume and promote further analyses, data management policies that promote data sharing, databases and software will be needed that are designed for secure and integrated compilation of phenotypic, genotypic, and environmental data that can be interrogated to reveal regulators of complex traits.

Precision breeding will integrate advanced technologies into production systems and provide feedback to selection programs. Technologies will be advanced so that deep phenotyping data can be transmitted in real time to researchers and producers. Precise phenotypes will lead to identification of genetic components for use in animal production and selection as well as for control of infectious diseases and selection for food quality traits. Leveraging preexisting data will help identify indicator traits for genetic evaluation; industry input will be essential. This has implications for improved health of animals and humans and prevention of pathogen transmission between the two (One Health^12^).

#### Expected Impacts and Deliverables

Phenotype collections will be expanded through the development of new technologies and expansion of existing technologies (e.g., wearable tape, sensors, cameras) to efficiently record data. For example:

✓ New sensors, methods, and software will be developed for collecting precise (detailed) phenotypes and prioritizing that information for larger-scale uses based on potential impact. Phenotypes will be developed that can be incorporated into genetic improvement programs.
✓ Access to data storage to facilitate transfer to end users will be expanded and improved.
✓ Economic data on the value of traits indexed for selection will be collected.
✓ Industry professionals (producers, veterinarians) will be trained on the value of novel monitoring for collection of basic data to improve genetic selection and management decisions.
✓ Extensive health and activity monitors will be developed to follow individuals and groups of animals under controlled conditions to identify critical phenotypic traits for later field applications with large numbers of animals.

#### Resources Required

Better collaboration between animal scientists and engineers capable of designing a variety of sensors is needed. Rural broadband will facilitate expanded use of data collected on farms. Other management uses of the collected data beyond genomic analysis will encourage farmers to undertake the expense and labor necessary to collect the needed data. Training will be available at all levels (students, faculty, producers, and consumers) for data analytics, management and visualization.

#### Partnerships

Advances in discovery science to make precision agriculture a reality for animal production will require interdisciplinary teams of scientists to address complex agricultural issues with state-of-the-art sensors, technologies, and methods to further animal agriculture. These interdisciplinary teams should include agricultural and data engineers, physiologists, and data scientists to achieve detailed phenotyping data and eliminate subjective evaluations. Animal scientists will need to engage engineers and software developers to design inexpensive, simple, and sturdy phenotyping devices that are minimally invasive and provide quantitative data to researchers, producers, and animal health experts. Programmers will need to work with animal breeders to develop ways to access detailed phenotypic data and design applications for genetic selection and management decisions.

### Harnessing the Microbiome to Improve the Efficiency and Sustainability of Animal Production

#### Vision

Identifying and tracking the symbiotic and pathogenic microbial components of animal production systems will enable the development of new tools and approaches that improve animal health, welfare, and production efficiency.

#### Current State of the Art

The important role of microbial populations (including bacteria, viruses, archaea, protists, and fungi) in animal health, production efficiency, and food safety has been suggested and confirmed in some cases. Throughout its life, an animal constantly tolerates, nurtures, and rids itself of different microbial communities on and within different body tissues. Many of these animal-microbial relationships comprise a mutualism that improves animal production or health; however, there are also neutral and harmful relationships that can inhibit productive potential, cause disease, or allow animals to serve as vectors for human disease. A proportion of observed phenotypic variation in animals may be due to differences in microbial populations. It has been proposed that partitioning of microbial components (Mi) to genetic or environmental terms of the standard P = G + E (Phenotype = Genetics + Environment) model may result in a modified model (P = G + E + Mi).

Microbiome analyses currently fall into two types. The first type of analysis surveys variation within ribosomal RNA genes to arrive at an assessment of the microbes that are present. Sequence variation within these genes provides an assessment of the genera present but it does not provide enough information to allow an assessment of microbes at the species level. The second type of analysis surveys the genes present within a microbial community to arrive at the biochemical activities that are present within the microbiome. This is useful in elucidating the biochemical and metabolic pathways that may exist within the system. One disadvantage with this type of analysis is that it is not always possible to predict the biochemical function of genes from their sequence. Each analytical method has advantages and disadvantages depending on the desired purpose, and further exploration of additional methods is needed.

#### Advancing the State of the Art

The symbiosis or dysbiosis of production animals with their microbial communities must be understood through systems biology approaches that fully evaluate the contribution of the host, the environment, and microbial communities to the target phenotype. This will require generating the following baseline resources:

• Comprehensive and longitudinal surveys of microbial nucleic acids in numerous locations in the host and environment;
• Standards for measurement tools, resources, and methods used to characterize microbial content and the effect of individual microbes on the tissue microbiome community; and
• Reference genomes for symbiotic, pathogenic, and transient microbial flora in the system.

##### Microbial genomes, transcriptomes, and metabolomes

Developments in technology during the next decade can reasonably be expected to support genome and transcriptome assemblies of many microbial inhabitants of animal tissues and fluids, providing important knowledge in gene content and metabolic capability. This in turn will support systems-based approaches toward model microbial activity and microbiome community development in the system. Metabolomic analyses will be needed to assign or confirm the assignment of function to some of these genes.

##### Host genome-microbiome interactions

A better understanding of host genome-microbiome interactions will be necessary for developing microbiome-based strategies for improving production. The extent to which the host genome is able to regulate the composition, and therefore function, of their microbiomes must be determined and accounted for in production systems.

##### Building the microbiome into animal production strategies

Once the baseline information of microbial community composition and function is in place, the inclusion of microbial data into animal phenotypic models will improve the accuracy of prediction in agricultural species. Development of rapid and inexpensive methods to characterize the components of an animal’s microbiome will allow analysis of entire populations. This will be necessary to provide the statistical power needed to identify key microbial signatures that influence animal disease incidence, nutrition, and production efficiency. This will also enhance food safety by developing processes for rapidly detecting zoonotic pathogen reservoirs. Finally, the development of interventions such as prebiotics and probiotics, vaccines, and holding facility treatments that benefit the microbiomes of specific animal populations should be investigated as ways to influence the microbiome to improve animal health and well-being, production efficiency, profitability, and sustainability.

##### Creating standards

One major impediment to microbial data interpretation is the current lack of a single, unifying set of standards for data collection and analysis. The creation of these standards is likely to promote meta-analyses of numerous contemporary microbial surveys. These types of cross-study analyses have been useful in the determination of fundamental constants and variables in other fields of science and are likely to advance the understanding of microbial system dynamics in general.

#### Resources Required

Investment in computational resources and infrastructure is needed. Given that microbial systems consist of many genetically distinct organisms competing/cooperating for resources, there is a need to improve computational resources that enable the rapid interrogation of microbial functional genetic elements and the comparison of microbial DNA sequence among different taxa. The creation of additional resources specifically devoted to the storage, dissemination, and analysis of agriculturally relevant microbial data would assist in this goal and may create an incentive for new microbiologists to mine these data sources for biological insights.

#### Expected Impacts and Deliverables

Over the next decade technologies will be developed that for the first time will include the microbiome in strategies to optimize animal production. Advances in microbial system modeling will result in improvements in animal health and welfare and many aspects of production. Identifying environmental and symbiotic microbial populations that promote animal health and welfare is a likely outcome that will convey social value and benefits to production. By reducing the need for the use of antimicrobial compounds to treat disease, it may be possible to reduce the likelihood that agricultural species could serve as reservoirs for resistant strains of pathogens.

#### Partnerships

Given the complexity and interdependency of microbial systems, microbiologists will need to develop long-lasting partnerships with animal scientists, animal geneticists, immunologists, and nutritionists to best identify causal factors that underlie microbial interactions in animal model systems. Conversely, microbial data will need to be transformed to fit into the models of other agricultural scientific fields to improve prediction accuracy. While this cooperation is likely to be slowed by the need to develop new methods and techniques to better integrate data from microbial sources, the rewards are likely to be a synergistic improvement on model prediction accuracy in all involved fields.

## Infrastructure

Infrastructure as described here is a combination of genomics equipment, bioinformatics and cyberinfrastructure, genetic resources, and training. The following paragraphs list the four goals from the 2008 Blueprint and contain a brief description of the accomplishments and remaining challenges that provide the foundation for the current blueprint efforts. In the 2008 Blueprint, the first goal was to *develop genomic tools to connect genotype to phenotype and elucidate pathways of complex traits for all agricultural animal species.* The need for *comprehensive, high-resolution genome maps and assembled and annotated genomic sequences* was also outlined. Whole genome sequences for most agriculturally important animals are now available, although the quality of the assembly in each of the species varies widely and needs improvement.

**FIGURE 9:**
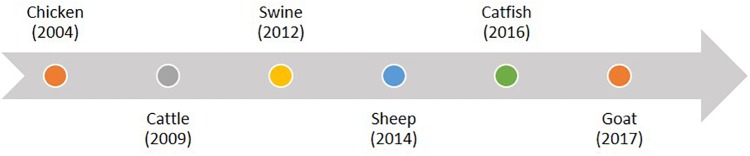
Publication dates for initial draft genome assemblies of major agricultural animals.

During the past decade sequencing technologies have improved significantly (higher quality reads, longer reads, paired end sequencing) while the cost of sequencing has decreased, resulting in new and/or improved assemblies for many animal species of agricultural importance (Figure 9). Software algorithms for genome assembly have also improved, so the most recent genome assemblies have much higher quality. The goat was the most recent livestock species to be sequenced in 2017 (Bickhart et al., 2017). The “*de novo* goat genome sequence is the most contiguous non-human diploid vertebrate assembly generated thus far using whole-genome assembly and scaffolding methods” (Worley, 2017). Using these newer sequencing platforms, significantly improved genome assemblies for many agriculturally important species are currently being generated. The latest cattle genome assembly (ARS-UCD1.2), swine genome assembly (version 11.1), and chicken genome assembly (version 6) were recently released. Genome annotation is based on transcriptome data, and gene names and functions are often based on similarity to human and mouse annotations, for which similar genes may have diverged in function. Genome annotation to better characterize the coding and non-coding genes and gene regulatory elements is at the preliminary stages and much more research in this area is needed. Genome annotation should not just include identifying the functional elements within the genome, but also adding functional information (standardized gene names, functions, interactions, expression information) so that these elements can be better linked to phenotypic data. Although obtaining genome annotation by comparing data with human and model species data has been helpful, it is critically necessary to develop annotations for each targeted animal species that are related to agriculturally important traits.

The second goal of the infrastructure discussion in the 2008 Blueprint was the development, deployment, and sharing of *national, comprehensive databases and the statistical and bioinformatics tools that integrate genomic, phenotypic, and experimental information for each species.* Along with reference genomes, vast amounts of genotype data from SNP panels are also now available, as are increasing numbers of individual animal genome sequences and information from RNA-seq experiments. These data are growing at an ever-increasing rate. However, integration of these data has not routinely occurred, and integration with performance and other phenotypic data has been rare. Furthermore, funding to support these developments and cyber-infrastructure has been limited. Lastly, data integration requires community standards and a commitment to data sharing.

The third goal in the section was the establishment of *centralized animal genetic resource populations that are deeply phenotyped and available to the research community*. In terms of deep phenotyping of a centralized resource population, the goal has not materialized. Thus, more effort is needed to link genotype and phenotype information in broadly available populations. However, the animal genomics research community has partnered with agricultural animal industries to address the need for large, commercially relevant populations for genomic analysis. In dairy cattle, genomic selection was initially applied to a population of bulls because historic DNA samples were available for a large assortment of these well characterized animals. This strategy led to rapid deployment of genomic selection in the United States. Several large consortia have leveraged resources and combined genotypic and phenotypic data to create resource populations that included a blend of animals owned by universities, private industry, and Federal agencies. Nevertheless, animal populations of the size needed for genomic analyses are expensive to maintain, and the ability of university, government, and other publicly funded organizations to shoulder this burden has eroded over time, reducing the discovery portion of genomic analyses.

The final goal in the infrastructure section of the 2008 Blueprint was to promote *education and training of students, scientists, and the public on genome-enabled animal science and opportunities that help prepare the next generation of researchers to work in interdisciplinary teams*. Animal genomics research occurs throughout the world; however, few of these programs integrate improvements in genome enabled quantitative selection in their research. Some groups offer training for students in the animal sciences to become adept at these types of analyses. At the same time, the sheer volume of data, and analytical methods to mine the data, have expanded faster than scientists can be trained. Thus, even though there are clear successes in training of future scientists in this area, the availability of trained individuals remains an important bottleneck in the development of future genomic research in food animals and in providing trained individuals who can help transfer these technologies to the industry.

The discussion that follows describes needed advances in infrastructure so that we can completely realize the potential of animal genomics. Advanced analysis tools will be needed to tailor genomic analyses to specific environments, optimize heterosis, incorporate epigenetic effects, and fit management to specific genotypes. To fully realize these advances, training in all these technologies will be needed for new scientists in these fields, but producers, veterinarians, extension specialists, and others will also need this training. In fact, data-intensive technologies, including genomics, will be so pervasive in the future of animal production that everyone involved in it will likely need training to adapt to it.

The genomic analyses and the automated phenotyping on which it will be based will require computing power and data storage and handling. Investments for improvements are needed so that the data generated can be used to support decisions that will make a real difference on the farm. Although gene-edited animal products do not yet exist in the U.S. marketplace, the potential use of these technologies to improve the production, health, and welfare of animals with a concomitant reduction in environmental footprint of animal production will eventually make them commonplace. Finally, as animals are bred to emphasize various traits, the ability to react to and recover from future challenges to animal production and health must be preserved. This includes preserving the current genetic diversity in species so that this genetic material is available if it is needed. A large portion of the genetic diversity that was once available in livestock, poultry, and aquaculture species has already been lost, and efforts are needed to prevent further genetic losses.

### Training the Next Generation of Animal Scientists

#### Vision

Effective implementation of genome-based methods to improve animal production will require a highly trained and skilled workforce that conducts research, commercializes new technologies, and more effectively communicates the nature and value of these methods to consumers and policymakers.

#### Current State of the Art

The training that graduate students in animal breeding, genetics, and genomics receive largely follows a traditional model based on coursework and performing independent research. Exposing undergraduates to opportunities in animal breeding, genetics, and genomics occurs primarily through courses offered in university animal science or agriculture programs. Students and professional scientists alike have little training in how to effectively communicate complex scientific topics to broad audiences.

#### Advancing the State of the Art

An infrastructure that offers specialized educational and training opportunities in academic, government and private sectors must be developed to ensure the Nation has a workforce that is highly trained and skilled in genomic and bioinformatic analyses. Education and training for undergraduate students, graduate students, veterinarians, industry professionals, and the public about genome-enabled technologies is paramount for the successful implementation of genetic improvement programs. Rapid technological advancements have made it feasible to obtain high-dimensional data sets (genotypes, phenotypes, sequence, etc.). Currently, few scientists are adequately trained to manage the computational complexities associated with analyzing and using these large data resources, while simultaneously understanding animal production systems. Therefore, enhancing education and training programs in data manipulation and genomics are essential if these new technologies are to yield their intended benefits. The next generation of animal scientists must be prepared to synthesize and integrate information, communicate effectively to broad audiences, and translate knowledge into practical applications. Scientists must effectively communicate the nature and value of their work so that consumers and policymakers can make informed, science-based decisions. This will require:

• *An integrated curriculum*: An interdisciplinary curriculum is needed at both the undergraduate and graduate levels that emphasizes data science and includes concepts of biology, microbiology, mathematics, statistics, computer science, and engineering.
• *Hands on training and mentorship:* Experiential learning opportunities must be created, and innovative training methods be developed by engaging and supporting industry professionals and government scientists to contribute to graduate and undergraduate education.
• *Recruitment*: Recruiting efforts must be enhanced to attract students from diverse backgrounds into computer science and engineering, and they must be encouraged to pursue employment in animal agriculture.
• *Communication*: Training must be provided to students in social science for critically evaluating information and effectively communicating with diverse audiences.

#### Resources Required

A centralized information repository is needed where training resources and opportunities can be collected and made available to students and university faculty, including online resources. Support for students and commitments from host institutions must be established, including incentives that motivate the development of mentoring programs for students from within and outside agriculture to work in various areas of animal production.

#### Expected Impacts and Deliverables

The result of this effort will be a cadre of well-trained and adaptable students who are prepared to fill positions in academia, industry, and government, and who can communicate about and apply emerging technologies to improve the efficiency of animal production systems.

#### Partnerships

Training the next generation of animal scientists who are capable of effectively communicating to other scientists, policymakers, and the public will require training and mentorship from experts in a wide range of disciplines, including biology, microbiology, data science, computer science, engineering, and communications. While most advanced training occurs within universities, industry and government sectors must also embrace the role of training the next generation. Scientists from the biomedical and veterinary communities should also participate in training and mentorship activities.

### Developing Advanced Genomic Tools, Technologies, and Resources for Agricultural Animals

#### Vision

Developing and applying rapidly evolving suites of genome-based tools and resources for all animal species that facilitate discovery science and lead to technological advances that will improve animal production.

#### Current State of the Art

Genome assemblies provide the underlying reference for genome-wide, discovery-driven experiments. Complete high-quality assemblies are now available for many animal species of agricultural interest. Advances in technology allow researchers to go beyond a single reference assembly and compare assemblies across breeds or varieties; however, computational methods for representing and comparing them are still in development. Furthermore, advances in computational analytics applied to genome assembly have allowed selection of an F1 of divergent parents as a reference animal for genome sequencing. This shift in methods means that two high-quality genome assemblies can be generated from a single animal, supporting immediate comparison of genomic variation between lines, subspecies, or species where interspecific crosses can be made.

Reference assemblies are necessary, but the full power of genomic analysis requires additional data to support annotation of the genome. An international consortium has been formed for the Functional Annotation of Animal Genomes (FAANG) effort (Andersson et al., 2015; Tuggle et al., 2016) to provide standardized methods for annotation data generation and analysis in a “gene-centric” effort. Some species have requisite data that exist to perform this annotation, but there may not be a concerted community effort. Beyond simple annotation of exons and introns, and cataloging relative expression levels, annotation includes regulatory elements (e.g., promoters, enhancers, microRNA targets) and base modifications associated with epigenetic activity across tissues and time points. Histone methylation and chromatin conformation assays also provide an additional level of annotation to characterize genome-level control points. An understanding of the interplay of regulatory elements, coding and non-coding RNAs, and chromatin states and how they affect the eventual production and characteristics of proteins and metabolites is now possible. These are much more expensive and less commonly performed than cheaper nucleic acid analyses, adding another layer onto gene-centric annotation. Finally, annotation at the level of gross tissue anatomy overlooks the variation inherent in different cell types within tissues; for example, different cell types are present in the blood. Emerging techniques in cell sorting and single-cell genomics are expanding opportunities to add precision to gene-centric annotation efforts.

Economical whole-genome sequencing technology supports efforts to identify single nucleotide, structural, and copy number variants across and within breeds, and supports quantitative genetic methods to identify, account for, and select variants with effects on phenotypic traits of importance. These population-wide screens and QTL/GWAS associations with phenotype require databases to document/display them in ways that facilitate discovery and testing of hypotheses. This includes both experiments in species where application of genotyping for selection is practical, and experiments resulting in direct release of improved germplasm to industry. Currently, most population genotyping is performed by genotyping arrays based on single nucleotide polymorphisms (SNPs), and this will likely remain an important method in the near term; however, cost reductions in whole-genome sequencing have begun to make comprehensive sequencing a feasible approach.

Transcriptomic analyses performed using either microarrays or sequencing are currently available to provide valid measures of expression of genes in specific tissues in a single experiment. To a much lesser degree, it is currently possible to obtain valid measures of large numbers of proteins and metabolites in the same tissues; however, these methods produce limited results because for the most part only the most abundant molecules can be measured. Using genomes as references, transcribed genes and proteins are easily identified and quantified, but methods to identify metabolites remain in development. Once these measurements are performed, analysis methods based on gene ontology or metabolic pathways can often provide clues to understand the enormous number of individual differences that occur in these data sets, but these methods are by no means comprehensive, and better analysis strategies are needed.

Current technologies generate a large amount of data, and storage of these data while maintaining access to these data by the community is an ongoing challenge. The National Agriculture Library has begun to develop the capacity to perform this role for the agricultural community.

#### Advancing the State of the Art

Advances in sequencing technologies, information sharing, and analysis algorithms will support the development of critical genomics infrastructure that supports discovery science and applications in the commercial sector.

##### Develop pan-genomes for agricultural animals

The human reference assembly does not represent any existing individual genome. Instead, it is a conglomeration of multiple genomes representing a group of individual genomes. Other species reference genomes generally represent a single individual, usually selected to be inbred when possible. A more suitable goal for a reference genome is to include all genomic segments that exist in all representatives (breeds, populations, etc.) within the species, termed the “pan-genome.” This resource is useful for insuring that all “re-sequencing” reads have a reference position and can be assessed for variation in the context of annotation. All animal species of agricultural importance should be characterized by pan-genome assemblies that, as with the human genome, provide full annotations of genomic features (Paten and Novak, 2017). Enhancing nucleic acid work with protein-level and metabolome-level techniques is also a major goal and implementing single-cell and functional genomic methods such as gene editing will enable enhanced granularity of resolution for genome annotation.

##### Genomic diversity in domestic livestock compared to wild progenitors

An important aspect of characterizing and cataloging genomic diversity in domestic livestock will be the comparative genome sequencing of wild progenitors (e.g., aurochs for cattle, wild boar for pigs, mouflon for sheep etc.). For some species, where the wild progenitor is extinct, this will involve paleogenomics. In particular, it will be important to sequence paleogenomes from centers of origin where animals were likely first domesticated (e.g., the Fertile Crescent). As sequencing technologies advance, this will become more straightforward. It is already possible to characterize paleogenomes from multiple ancient wild and early domestic populations (Wales and Melis, 2018).

Currently, most surveys of paleogenomic information from livestock and other domestic animals is focused on relatively broad, but shallow population genomic comparison to modern populations (MacHugh and Larson, 2017). However, as sequencing depths increase, high-resolution genomic information from extant congeners, wild ancestors and early domestic populations (i.e., Neolithic populations across Eurasia) can be integrated with population genomics data from modern domestic populations and functional genomics data for the following purposes:

(1) Fully characterize genomic diversity in a domestic species, both spatially and temporally through time. This will facilitate identification of important genomic variants relevant to production, health and welfare traits. This will be relevant for genome-enabled breeding and for providing targets for genome editing.
(2) Identify adaptive introgression of valuable alleles from wild ancestors (e.g., aurochs into early domestic cattle) and extant congeners (e.g., wild boar into domestic pigs). This will be relevant for genome-enabled breeding and for providing targets for genome editing.
(3) Comparison of genomic information between domestic populations and extinct progenitors or extant congeners will provide information on genomic evolution as a consequence of the domestication process. This will provide valuable information concerning the domestic animal behavior and welfare.

##### Catalog genetic variation

In addition to multiple annotated genomes that encompass the diversity of each species, databases of sequence variants need to be maintained and/or created. Additional databases that currently identify QTLs and place them in context with local variation segregating in the population(s) used to identify them can help identify underlying functional variation. It is reasonable to expect that databases of known functional variants affecting many phenotypes can be developed for major species, which will greatly enhance genomic selection for trait improvements. Concordant development of defined phenotypes and standardized collection methods would facilitate this advance. Component phenotypes of important traits related to animal health, feed efficiency, and food safety should be targeted because these are more difficult for producers to collect in useful ways. Reference databases of the complete genome sequences for prokaryotic and eukaryotic microbes in animal production environments should be created and maintained to support microbiome studies. Sampling to encompass eventual or concomitant analysis of microbiomes with other phenotype/genotype collections should be encouraged.

##### Use functional genomics

Better methods to provide comprehensive proteomic and metabolomic analyses on low abundance molecules are needed. In addition, better analysis methods and software are needed to extract information from the increases and reductions in gene expression and protein and metabolite abundance measures. Equipment and software are also needed to integrate these diverse information streams to improve understanding of biological systems. These abilities will be an important step toward carrying out fine-scale studies that result in the discovery of molecular mechanisms impacting the phenotypes of economically important traits.

#### Resources Required

Many important studies that can contribute to genome annotation require access to a common set of tissues that can be used to perform multiple assays and can be examined to determine how genome sequence variation affects structure and function. A useful goal in this regard would be the establishment of broadly available curated tissue resources and cell lines, organoids, and ultimately phenotyped populations to contribute some consistency among experiments. Large-scale efforts that use reverse genetic methods will require defined and accessible information such as comprehensive guide RNA libraries for gene editing. As technologies advance, swapping large genomic segments with desired genomic variations may require the availability of large-insert bacterial artificial chromosome libraries.

As previously indicated, another key resource is computational infrastructure that can hold and provide ready access to large datasets and databases, and analytical software that is computationally efficient and user friendly. Scientific and funding agencies should recognize that a hallmark of genomic research has been the emergence of disruptive technologies that can quickly change the research landscape. Predicting these breakthroughs is difficult, but flexibility to incorporate these technologies “on the fly” into planned and ongoing activities/funding calls should be explicitly recognized and a focus of planning.

#### Expected Outcomes and Deliverables

Fully annotated animal genomes will contribute greatly to genomic selection strategies. Functional genomics technologies will reveal numerous novel traits that could be used to dissect complex and poorly heritable traits and improve selection progress. Better functional genomics analysis strategies will improve our understanding of complex biological systems, resulting in management strategies beyond genomic selection that can be used to improve economic traits.

#### Partnerships

The entire agricultural community, including the public and private sectors, contribute to and benefit from the generation of fundamental genomic methods and resources. The United States has led many of these efforts, but coordination with international partners is essential. The animal genomics community must partner with other genomics communities (e.g., plant, biomedical) to optimize the use of genome information; must partner with livestock, poultry, and aquaculture producers who have access to diverse germplasm and breeding programs for implementation; and must partners with researchers and companies who advance DNA genotyping, sequencing, and analytical technologies.

### Creating Big Data Tools and Infrastructure for Animal Production

#### Vision

Developing state-of-the art bioinformatics databases and methods will provide the research community with the computational infrastructure it will need to process, analyze, share, and reuse genomic and phenotypic data.

#### Current State of the Art

Modular reuse of bioinformatics methods allows genomics data to be processed rapidly. Many techniques and software systems are available to genomics researchers for the analysis of sequence/genotypic data. They can be combined in different ways to facilitate data analysis to meet various goals. However, many bioinformatics methods work only on the computational platform where they were developed. The vast majority of existing functional genomics data is used only in the project in which it was generated, whereas genomic re-sequence data and animal genotype data are routinely used several times. The long-term stability of animal genome databases requires a solution (e.g., the NCBI dbSNP is no longer available for inputting livestock genetic variant information). The National Agriculture Library is developing the computer infrastructure to fulfill this role for the community, but further support is needed.

#### Advancing the State of the Art

Animal scientists must have access to critical infrastructure to apply state-of-the-art technologies to animal production. Modern infrastructure is needed to integrate real-time genotypic, functional genomic, and metagenomic data to predict animal phenotype, and needs the capacity to reuse large and complex data sets. This will require the following:

• *Computational capacity*: Faster bioinformatics tools must be developed to analyze data (e.g., genetic evaluation, variant association testing, DNA/RNA sequence analysis proteomic, metabolic, and microbiome data).
• *Usability*: Bioinformatics data, software and methods must be available for use across a variety of computational systems.
• *Metadata standards*: Standards for metadata (data providing the context of the stored data such as animal, tissue, assay, format of storage used, etc.) are needed to better document research data.
• *Databases*: Better animal genome databases are needed to facilitate genomics research and translation to industry.

#### Expected Impacts and Deliverables

Investments in big data tools and infrastructure are expected to result in the following impacts and deliverables:

✓ Analyses of large integrated data sets with greater numbers of data varieties at an improved velocity to better inform breeding and management decisions.
✓ Reuse of genomics data will accelerate genomics research and open new areas of research to facilitate genomic improvement of animals.
✓ Expansion of data storage will facilitate transfer to end users.
✓ Better use of resources (e.g., human and computational).
✓ Training will be available to all users (students, faculty, producers, veterinarians, consumers) for data analytics and visualization.
✓ Data analysis pipelines will be commonplace and will be used routinely for genomic and non-genomic research and application.
✓ Improved databases will provide ready access to genomics data.

#### Partnerships

Collaborations with large database communities (e.g., NCBI, Ensembl, Encode) are critical for improving infrastructure for animal species. Data and computer scientists are needed to develop, improve, and optimize methods, techniques, software, and databases. Animal breed associations, companies, and producers must have access to data and provide feedback on the usefulness of that data.

### Advancing Biotechnology to Improve the Sustainability and Efficiency of Animal Production

#### Vision

Implementing animal production biotechnology as it has been implemented in crop production will facilitate discovery science, accelerate genetic improvement, and enhance strategies to optimize animal production.

#### Current State of the Art

Biotechnologies such as genomic selection are often combined with advanced reproductive techniques such as cloning, embryo transfer, and artificial insemination to accelerate the rate of genetic gain in animal breeding programs. Genetic engineering can be defined as the manipulation of an organism’s genes by introducing, eliminating, or rearranging specific genes and/or genetic variation using the methods of modern molecular biology, particularly those techniques referred to as recombinant deoxyribonucleic acid (rDNA) techniques. However, genetic engineering has resulted in only a single approved application for an animal to be used for human consumption, the fast growing AquAdvantage Atlantic salmon (FDA, 2015), even though the methodology was developed almost 30 years ago. Researchers have developed useful applications using genetic engineering to develop experimental breeds of animals that have increased disease resistance, utilize feed more efficiently, produce less waste, and produce more healthful products. However, the high regulatory costs and unpredictable timeline of producing such animals, combined with opposition from groups opposed to the use of genetic engineering in agricultural production systems, has effectively precluded animal breeders from using this breeding method in animal genetic improvement programs. This is in stark contrast to its use in plant agriculture, where in 2017 genetically engineered crops were planted by up to 17 million farmers in 24 countries on a total of 189.8 million hectares (469 million acres) (ISAAA, 2017). This is equivalent to almost 20% of the total land area of the United States (937 million hectares).

This situation has the potential to change with the development of new gene editing technologies. Specifically, gene editing techniques have the capacity to reduce threats from current and emerging zoonotic pathogens; reduce required levels of production inputs (e.g., space, water, energy, or feed); alleviate animal stress and distress from disease, heat stress, the production environment, and other factors; and improve growth efficiency, animal well-being, nutrition, and product quality. The current methodologies of genome/gene editing using site-directed nucleases and base editors (e.g., CRISPR/Cas9) facilitate the production of small edits such as single base changes and small insertions/deletions. However, these technologies also enable larger copy or paste-type modifications for the insertion or deletion of larger gene fragments. The recent National Academies of Sciences, Engineering, and Medicine study “Science Breakthroughs 2030: A Strategy for Food and Agricultural Research” identified “the ability to carry out routine gene editing of agriculturally important organisms” as one of the five most promising scientific breakthroughs that are possible to achieve in the next decade to increase the U.S. food and agriculture system’s sustainability, competitiveness, and resilience (NAS, 2015, 2018). To prevent gene editing from meeting with the same opposition previous genetic engineering methods encountered, the National Academies’ report further recommends using social science findings about factors influencing the acceptance and adoption of scientific information to develop educational and communication materials about this technology for producers and the public.

Perhaps the most ambitious goal stated in the report is a “10-fold increase in the rate of genetic improvement” in livestock, poultry, and aquaculture populations by 2030. This improvement will require merging modern biotechnologies, genomic information, quantitative genetics, and advanced reproductive technologies. Such a step change could be envisioned via *in vitro* breeding techniques that are of particular importance to species with a long generation interval (e.g., cattle).

**FIGURE 10:**
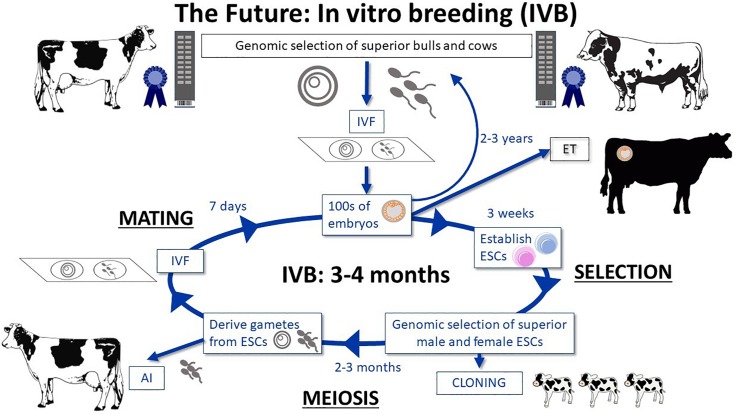
Efficient isolation of pluripotent embryonic stem cells (ESCs) from cattle embryos allows the development of *in vitro* breeding schemes based on an embryo-stem cell-gamete cycle, including an intermediate genomic selection to provide directional selection of genetic progress. If such a scheme could be accomplished, it would significantly decrease the generation interval and allow for increased selection intensity leading to accelerated genetic progress. IVF, *in vitro* fertilization; ET, Embryo transfer. Image from Van Eenennaam (2018). Reproduced with permission from the author’s entry in the *Encyclopedia of Food Security and Sustainability*, Ferranti et al., (2018).

This 10-fold goal could be achieved by reducing to practice *in vitro* meiosis and embellishing upon ideas proposed more than 25 years ago by Georges and Massey (1991). There the authors postulated the concept of harvesting oocytes in meiosis to reduce the generation interval. This could be further reduced through *in vitro* meiosis, effectively removing the biological limits on the generation interval. Researchers working in mice have been able to derive both sperm and eggs from embryonic stem cells (ESCs) (Hikabe et al., 2016; Zhou and Wang, 2016). ESCs have recently been isolated from cattle (Bogliotti et al., 2018), raising the possibility that this method or technique might be applied to livestock species. Figure 10 shows the amount of time it might take to perform a round of “*in vitro* breeding” for cattle. Obviously, this may have dramatic effects on the rate of genetic gain in terms of reducing the cattle generation interval from 2–3 years to 3–4 months, offering a way to dramatically reduce the generation interval and conceptually enable a tenfold increase in the rate of genetic gain.

Other applications of gene editing have the potential to dramatically reduce the lag in genetic merit that typically exists between the seedstock sector and the commercial sector. Knocking out a specific gene or genes (e.g., *PDX1* for sheep pancreas development; Vilarino and Rashid, 2017) required for the development of a particular organ can create a genetic “developmental organ niche” in that animal, the host. Pluripotent stem cells from a donor can then be used to colonize that vacant niche in the host through stem cell transplantation or blastocyst complementation (i.e., allogeneic donor cells are combined with host blastomeres to form a new embryo).

Of particular interest to the livestock industry is the possibility of being able to generate germ cells from high-merit donor animals exogenously in the gonads of otherwise sterile host animals, thereby expanding the availability of gametes from genetically desirable dams and sires. It was recently reported that sterile testes from *NANOS2* knockout boars were able to harbor donor-derived spermatogenesis following transplantation and regeneration of wild-type spermatogonial stem cells (Park, 2017; Oatley, 2018). Exogenous germ cells have also been generated in the sterile ovaries of *NANOS3* knockout cattle that were produced through blastocyst complementation involving the microinjection of donor (*NANOS3*^+/+^ Holstein) blastomeres into host (*NANOS3*^−/−^ Wagyu) morulas (Ideta et al., 2016).

The application of this research could provide an alternative to artificial insemination by allowing the use of environmentally adapted surrogate sires to deliver superior genetics through natural service. Beef and sheep industries would be the most obvious beneficiaries of these developments, given the logistical difficulties of implementing artificial insemination when animals are managed under extensive range conditions. The dairy industry might also benefit because top sires are often unable to produce sufficient amounts of semen to address demand. Moreover, for developing countries, the elite genetics of locally adapted cattle breeds could be more easily distributed using natural service, rather than the more technically challenging and resource intensive artificial insemination (which requires liquid nitrogen, synchronization of hormones, etc.).

#### Advancing the State of the Art

Genome writing and/or editing holds great promise as a genetic improvement tool. Implementing these biotechnologies will require the steps outlined below.

##### Expand implementation of biotechnology

Currently, the application of this technology by stakeholders is severely underused but could be improved by research progress or breakthroughs in the following areas:

(1) Characterizing genes and sequence variation that directly affect gene function and expression of phenotypes;
(2) Improving efficiency, specificity, and fidelity of site-directed nucleases (SDNs) and base editors;
(3) Identifying or developing novel methods for genome writing and/or editing;
(4) Improving the efficiency of producing live, viable animals from assisted reproductive technologies that enable genome editing (including but not limited to *in vitro* fertilization and cloning);
(5) Characterizing mutagenic and epigenetic events caused by any of the technologies associated with genome modification;
(6) Adapting editing technologies to other species (birds, fish, and emerging cultured species); and
(7) Exploring opportunities to accelerate genetic improvement through the integration of genomic information, advanced breeding technologies, and precision breeding methods.

##### Extend genotype to phenotype

While work continues to extend sequence variation discovery by building better genomic tools for genetic analyses across many food animal species, additional efforts should be initiated to test genotype to phenotype (G2P) effects of important candidate quantitative trait nucleotides (QTNs) already hypothesized. Understanding QTNs in differing genetic backgrounds and across species will reveal new insights into understanding the relationship between genotype and phenotype. New efforts to comprehensively interrogate food animal genomes using site-directed nuclease mutagenesis of host genes that interact with pathogens should be initiated to discover modifications that create resistance to disease. Similarly, for genes that are known to have many variations in nature, the entire sequence space should be investigated *in vitro* and *in vivo*. These efforts should not be limited to specific technologies but should include synthetic biology as well as *in vitro* and *ex vivo* directed evolution that is confirmed in live animals. Together, these efforts will improve our knowledge of how the genotype of an animal can be precisely manipulated to optimize animal performance and well-being based on the production environment and management inputs.

Ultimately, the future of these technologies requires an attitudinal change by scientists in how they think about the applications. Applications should include basic scientific questions and production, safety, palatability, and animal welfare issues. In addition, evaluation of risk/novel hazards should be evaluated but should be limited to those cases for which an actual biological basis for concern can be named or described.

##### Agricultural genes and phenotypes

Special attention should be given to genes that are present in agricultural species and cannot be directly investigated in the mouse due to differences in physiology, phenotype, or gene structure. This area of investigation should be expanded through partnerships with other research organizations in those cases where the mouse is not an appropriate model for a parallel question. This will include:

• Expanding, refining, and/or developing methods to extend genome modification capabilities to other agricultural species (for example turkeys, fish, mollusks, etc.).
• Expanding, refining, and/or developing methods to improve the efficiency of assisted reproductive technologies and increase the efficiency of genome modification in all species.
• Characterizing the unintended consequences of current editing methods in comparison to conventional breeding methods with regard to both genetic and epigenetic effects.
• Confirming that specific sequences or modifications impact phenotype in predicted ways (confirming potential causative variation and affirming lack of biologically relevant off-target modifications).
• Developing tools and reagents that facilitate many avenues of research (e.g., lineage marked animals for cell sorting, immortal or primary cell lines or other *in vitro* techniques).

#### Resources Required

Currently, researchers need animals that are designed to query specific biological questions. To address these questions (e.g., are the direct effects of genes endogenous or exogenous to the host? What is the variation observed within and between species?), and to study novel/rare combinations of alleles, genetically edited/modified animals must be available for the research community.

Because finding answers to these questions is inhibited by infrastructure limitations, it is necessary to increase our ability to perform single-cell interrogation, gene editing, knockout, and overexpression experiments.

#### Expected Impacts and Deliverables

Developing and expanding the use of animal biotechnologies in agricultural animal research and production will:

✓ Provide better tools for investigating the biology of economically important traits;
✓ Identify and quantify potential risks and benefits associated with the implementation of biotechnologies in animal breeding schemes within the context of those posed by conventional breeding programs;
✓ Provide an additional tool for biological approaches to improving animal production, well-being and product quality;
✓ Produce gene-edited animals that have improved production, welfare, and health traits to meet the needs of consumers of animal products; and
✓ Accelerate the rate of genetic gain toward targeted breeding goals in animal breeding programs.

#### Partnerships

Collaboration between genome editors/writers and other researchers aiming to address all aspects of animal production is necessary to achieve successful evaluation and implementation of biotechnologies. Community education is needed to promote the importance of these editing tools in producing food animals with superior traits.

### Characterizing and Preserving Genetic Diversity for the Future of Animal Production

#### Vision

Characterizing, sequencing and preserving genetic diversity representing commercially important animals, their wild relatives, rare breeds developed for specific traits, and specialized research populations will enhance discovery science, provide a resource of genetic diversity that will enable breeding strategies to respond to changing priorities, and allow recovery from disease outbreaks or other occurrences that would reduce the genetic diversity of animal populations.

#### Current State of the Art

Commercial animal breeds have been selected for very specific performance features and therefore represent a limited gene pool. There is considerable genetic variation in other breeds that is not present within these limited production breeds and this variation is not well documented or understood. Standard breeds, landraces, and heritage breeds are highly valuable as sources of untapped variation. Now that genome reference sequences and the tools to identify various types of variation are available for each of the major livestock, poultry, and aquaculture species, it is time to explore the diversity present within each species. With the continual changes in the production environment, including reductions in available disease treatments, changing environmental characteristics, and changing production demands, understanding this variation will lead to the identification of candidate genes and variants that could be introduced to commercial populations through introgression or genome editing to enhance performance.

Conservation of genetic material with potential commercial value is of great interest to commercial breeders. If the direction of genetic selection drives populations to an undesirable phenotype, or if market demand develops for a different desired phenotype, the ability to go back multiple generations and access the larger range of genetic diversity to change the direction of genetic selection would be of great value. In addition, scientists cannot predict with absolute certainty which genetic traits will be of value in the future, and where that variation may currently reside. Therefore, it is not only important to conserve commercial germplasm, but it is crucial to preserve and develop assistive reproductive technologies for closely related species that currently have less agricultural value.

The conservation of heritage populations, both randomly selected and control populations, would allow breeders to access previous genetic information and provide increased flexibility in fulfilling variable market needs. The high costs of maintaining unselected or currently underutilized lines inhibit the maintenance of these populations within commercial breeding facilities. Moreover, all these technologies will be important for saving endangered species. Ruminant genomics will inform the rhinoceros genome, for example, and likely help in efforts to reproduce the endangered white rhinoceros.

The United States maintains considerable stocks of genetic material from a wide variety of animals (Supplementary Appendix 6 and Table 2), however there have been considerable documented losses of genetic diversity over the past four decades (FAO, 2015). This diversity is at very high risk of being lost due to lack of organization, documentation, and communication among breeders. This diversity is underrepresented within the existing cryopreserved materials and is generally greatly underrepresented in any studies on genetic variation. Heritage and rare breeds often have unique phenotypes that are highly divergent from mainstream production breeds. Many of these breeds also have extensive historical records related to their breeding and maintenance. These breeds therefore represent populations with useful genetic information and may reveal novel genetic pathways such as those involved with production and adaptation traits. Some swine breeds, for example, are resistant to colibacillosis by virtue of a single gene mutation (Meijerink and Fries, 1997), and Blanco-Orejinegro cattle are resistant to infestation by *Dermatobia* spp. and also resist the effects of the bovine leukosis virus (Rodrigo Martínez, 2010).

**Table 2:** Preserving characterized genetic diversity for important animal species in National Animal Germplasm Preservation Program (https://nrrc.ars.usda.gov/A-GRIN/main_webpage_dev/ars?record_source=US).

Common Name	Number of Animals	Units of Germplasm	Phenotypic Data	Molecular Data
Aquatic Freshwater Fish	6,813	95,664		
Aquatic Invertebrates	218	7,485		
Aquatic Marine Fish	15	823		
Beef Cattle	9,773	241,410	Yes	Yes
Bison	75	1,631	Yes	
Chicken	1,936	14,752	Yes	
Dairy Cattle	7,187	253,113	Yes	Yes
Elk	4	340		
Goat	490	10,579	Yes	Yes
Honey Bee	3	21		
Horse	20	150	Yes	
Nematode	19,169	19,169		
Pig	1,537	217,703	Yes	Yes
Screwworm	10	19,350		
Sheep	2,832	65,093	Yes	Yes
Turkey	242	624	Yes	
Yak	4	111	Yes	
**TOTAL: 17**	**50,328**	948,018		

Unique characteristics of different animal populations make them useful in understanding the effects of genetic variation on variable and unique phenotypes and for understanding novel genetic pathways for characteristics that could be adapted because of their potential commercial value. Research lines, due to their specific and well-documented breeding histories, provide a resource for insights into gene function and expression. Specially selected lines are useful for understanding the underlying genetic architecture for specific traits, such as those divergently selected for body weight, specific immune response, prolificacy, and seasonality of breeding. Comparisons between genetic lines of animals preserved intact for decades and their modern highly selected equivalents have greatly informed our understanding of the underlying changes in genetic architecture due to long-term selection. Genomic techniques are now available to study the genetic variants that separate these selected lines and to gain information to improve our understanding of gene function, functional pathways, and phenotypic effects.

Technologies for cryopreservation of germplasm exist for many agriculturally relevant animal species, but not for all the species that are important to preserve (FAO, 2012). Moreover, for many species these technologies are inefficient. Different species react differently to cryopreservation methods. Long-term preservation of semen, embryos, gonads, and primordial germ cells in a deep-frozen state (cryoconservation) can provide a measure of insurance against the loss of genetic diversity, whether among or within a specialized line, breed, or species. The germplasm preservation methods are still underdeveloped for certain species (poultry and aquaculture) and *in situ* methods may be required for preserving certain high-value genetic lines until we have robust preservation methods developed. More research is needed in this area.

#### Advancing the State of the Art

##### Improve cryopreservation protocols

Species-specific protocols must be developed to the degree they can be routinely used to preserve sufficient germplasm for all agriculturally important animals. Further efforts are needed to understand the mechanisms of effective cryopreservation among domesticated species, including studies of the effectiveness of various strategies and tissue/cell sources for this purpose. Research needed in cryopreservation of gonads includes surgical techniques, preventing rejection of grafted tissue, and methods for depleting the recipient gonads to ensure exclusive production of donor gametes.

##### Characterize genetic diversity

Estimates for genetic and phenotypic diversity are lacking for many U.S. breeds, including rare breeds, landraces, select populations, and heritage breeds. Animals with extreme phenotypes, in addition to mid-range phenotypes, should be characterized and preserved. This type of information is needed to better identify unique sources of variation and to inform future conservation efforts. Likewise, collections of germplasm and tissues from diverse populations, including commercial breeds, must continue and be expanded. Phenotypic information is either incomplete or lacking for many rare breeds. The assumption, in the absence of such data, is that rare breeds have little to offer production agriculture. But the genetic profiles of Red Polled cattle, Spanish goat in the United States, and Bani buffalo of India demonstrate some rare and overlooked breeds have important phenotypic characteristics that make them sound choices for production agriculture.

##### Maintain genetic diversity

Little is known about the effective population sizes, population structures, and other characteristics of rare breed populations. A broad assessment of this diversity is needed to provide information on genetic relationships, variability, novel phenotypes, disease resistance, environmental adaptation, and other traits. Conservation programs cannot be developed without first documenting the variability that currently exists and then determining how this variability affects the phenotype. Only then can informed decisions be made with regards to appropriate conservation.

#### Resources Required

There has been a national contraction in research populations held by public institutions, which limits the research community’s ability to explore existing breed diversity outside of commercial settings. Conservation of unique genetic resources must be a national priority for animal agriculture. New models for how to study the existing genetic diversity within the research community need to be established, or else many of the unique lines will never be characterized or their value identified. A systematic approach to evaluating the existing diversity within agriculturally relevant animal species is needed and should be implemented before the diversity that exists in small private collections of living animals is lost. This approach, however, contains unique challenges, because cryopreservation of viable genetic material is in its early stages and has not been fully proven across breeds and laboratories.

#### Expected Impacts and Deliverables

Identifying, characterizing, and preserving genetic diversity for domestic breeds and for commercial, wild, and research populations will have the following effects and outcomes:

✓ Enhanced security for existing genetic diversity, ensuring it can be accessed in the future;
✓ Informed decisions on preservation/conservation of breeds/populations will be enabled;
✓ Valuable resources will be provided to support discovery science;
✓ Awareness of the unique characteristics of specific populations will increase; and
✓ Scientists will have easier access to unique well-characterized germplasm through sharing services.

#### Partnerships

Achievement of these objectives will require members of the animal genomics community to partner with heritage and breed associations, managers of valuable research and commercial populations, and members of the biomedical community who develop technologies associated with cryopreservation and reproductive technologies.

## Conclusion

The United States enjoys a strong heritage as an agricultural nation whose farmers continue to provide consumers with a consistently nutritious, safe, and abundant food supply. Because the Nation values technology and innovation, the animal genomics community is well positioned to address current and future agricultural challenges facing the planet. Over the last decade, USDA-funded scientists and their colleagues from across the globe used the 2008 Blueprint to develop an animal genomics infrastructure that facilitated novel scientific discoveries, some of which were implemented into commercial production and led to returns that far exceeded investment costs. The 2018 Blueprint will continue this trajectory based on new technologies, new insights into animal biology, and new genome-enabled strategies that improve various aspects of production. Ultimately, animal genome technologies will become part of mainstream agricultural production strategies used to improve animal health, well-being, production efficiency, and product quality in ways that meet the demands of growing global populations.

## Author Contributions

All authors listed have made a substantial, direct and intellectual contribution to the work, and approved it for publication.

## Conflict of Interest Statement

The authors declare that the research was conducted in the absence of any commercial or financial relationships that could be construed as a potential conflict of interest.

## Footnotes

1 https://www.asti.cgiar.org/brazil

2 https://www.asti.cgiar.org/india

3 https://www.asti.cgiar.org/china

4 https://www.animalgenome.org/share/meetings/Genome2Phenome

5 http://nap.edu/25059

6 https://www.uscdcb.com/wp-content/uploads/2017/09/CDCB-Health-Traits-FAQs-10_2017.pdf

7 https://goo.gl/S8zYJ6

8 http://sero.nmfs.noaa.gov/sustainable_fisheries/gulf_fisheries/aquaculture/documents/pdfs/genetic_requirements_guidance.pdf

9 https://www.animalgenome.org/cgi-bin/QTLdb/index

10 http://useast.ensembl.org/info/genome/variation/species/species_data_types.html#sources

11 www.encodeproject.org

12 http://www.onehealthinitiative.com/
