# Supplementary material for: Genome to Phenome: Improving Animal Health, Production, and Well-Being – A New USDA Blueprint for Animal Genome Research 2018–2027
Source: Front Genet. 2019 May 16;10:327. doi: 10.3389/fgene.2019.00327 (PMC6532451; doi:10.3389/fgene.2019.00327)

**Genome to Phenome: Improving Animal**

**Health, Production, and Well-Being**

***A New USDA Blueprint for Animal***

***Genome Research 2018–2027***

**Supplementary Files**

Contents

[Definitions 4](#_Toc527649180)

[Appendix 1: Steering Committee 10](#_Toc527649181)

[Appendix 2: Writing Teams 11](#_Toc527649182)

[Appendix 3: Workshop 13](#_Toc527649183)

[Appendix 4: Workshop Participants 18](#_Toc527649184)

[Appendix 5 Animal Genome Information 21](#_Toc527649185)

[Appendix 6 Animal Germplasm Preservation 24](#_Toc527649186)

## Definitions

**Artificial insemination (AI)**

The delivery of semen into the uterus of a female animal usually by injection with a syringe-like apparatus for the purpose of achieving fertilization and sexual reproduction.

**Alleles**

Variant forms of the same gene, occupying the same locus on homologous chromosomes, and governing the variants in production of the same gene product.

**Bioinformatics**

A field of biology concerned with the development of techniques for the collection and manipulation of biological data, and the use of such data to make biological discoveries or predictions. This field encompasses all computational methods and theories applicable to molecular biology and areas of computer-based techniques for solving biological problems, including manipulation of models and data sets.

**Biotechnology**

A range of techniques, including traditional breeding, that alter living organisms, or parts of organisms, to make or modify products; improve plants or animals; or develop microorganisms for specific agricultural uses. Modern biotechnology today includes the tools of genetic engineering.

**Breed**

A stock of animals or plants within a species having a distinctive appearance and typically having been developed by deliberate selection.

**Breeding Value**

The sum of gene effects of a breeding animal as measured by the performance of its progeny.

**Causal allele**

The functional allele that influences disease susceptibility and explains the observed associated allele.

**Chromatin/Chromatin structure**

The material of which the chromosomes of organisms other than bacteria (i.e., eukaryotes) are composed. It consists of protein, RNA, and DNA.

**Crossbred**

A crossbred plant or animal; hybrid.

**Cryopreservation**

The preservation of cells and tissue by freezing.

**Cyberinfrastructure**

Information technology systems that provide particularly powerful and advanced capabilities.

**DNA methylation**

Addition of methyl groups to DNA. DNA methyltransferases perform this reaction using S-adenosylmethionine as the methyl group donor.

**Dominance**

The phenomenon whereby in an individual containing two allelic forms of a gene, one is expressed to the exclusion of the other.

**Epigenomics/epigenetics**

The study of the mechanisms by which genes bring about their phenotypic effects.

**Functional annotations**

The process of attaching biological information to genomic elements.

**GBLUP**

A method that computes or imports a genomic relationship matrix and from that computes the “Genomic Best Linear Unbiased Predictor” (GBLUP) of additive genetic merits by sample and of allele substitution effects (ASE) by marker.

**Gene**

The fundamental physical and functional unit of heredity. A gene is typically a specific segment of a chromosome and encodes a specific functional product, such as a protein or RNA molecule.

**Gene editing**

Technologies that make specific, directed changes to the DNA sequence of an animal.

**Gene/protein expression**

The result of the activity of a gene or genes that influence the biochemistry and physiology of an organism and may change its outward appearance.

**Gene regulatory networks**

Interacting DNA-encoded regulatory subsystems in the genome that coordinate input from activator and repressor transcriptional factors during development, cell differentiation, or in response to environmental cues. The networks function to ultimately specify expression of sets of genes for specific conditions, times, or locations.

**Genetic gain**

The amount of increase in performance that is achieved through artificial genetic improvement programs.

**Genetic markers**

A phenotypically recognizable genetic trait that can be used to identify a genetic locus, a linkage group, or a recombination event.

**Genetic merit**

The ability of an individual parent to produce superior offspring with desirable traits as compared to other parents.

**Genetics**

Manipulation of the genetic makeup of an animal through either traditional (phenotypic) or genome-enhanced (tracking individual DNA variations among animals) selection, mutagenesis (random mutation of DNA sequence), or directed changes to specific DNA (gene editing).

**Genetic variation**

Genotypic differences observed between or among the species in a population.

**Genome**

The complete DNA sequence of an animal.

**Genome annotation**

The process of identifying the locations of genes and all the coding regions in a genome and determining what those genes do. An annotation (irrespective of the context) is a note added by way of explanation or commentary. Once a genome is sequenced, it needs to be annotated to make sense of it.

**Genome editing**

Genetic engineering techniques that involve DNA repair mechanisms for incorporating site-specific modifications into a cell’s genome.

**Genome selection/Genome-enabled selection**

A new method of improving quantitative traits in large plant-breeding populations that uses whole‐genome molecular markers (high-density markers and high‐throughput genotyping).

**Genome tools**

Tools to prepare or manipulate redundancy compressed genomic data.

**Genomics**

Technologies that characterize and manipulate the genome (DNA) of an animal.

**Genotyping**

Methods used to determine the specific alleles or SNPs (single nucleotide polymorphisms) of an individual.

**Germplasm**

The cells or tissues from which a new organism can be generated.

**Heterosis**

The adaptive superiority of the heterozygous genotype with respect to one or more characters in comparison with the corresponding homozygote.

**Metabolome**

The complete set of metabolites and endogenous small molecules involved in metabolism of whole cells. Metabolome varies with the physiological or developmental stage of the cell.

**Metabolomics microbiome/metagenome**

Analysis of the genetic material in a collection of organisms, usually applied to uncultured, mixed-species microbial communities in their natural environment.

**MicroRNA**

Small double-stranded, non–protein-coding RNAs, 21–25 nucleotides in length generated from single-stranded microRNA gene transcripts by the same ribonuclease III, Dicer, that produces small interfering RNAs.

**Nonadditive effects**

Sometimes one allele, the dominant one, masks expression by its partner, the recessive allele. The organism will display the dominant trait if it has either one or two copies of the dominant allele for that trait. The recessive trait prevails only if the organism is homozygous for the recessive allele (that is, if it has two copies of the recessive gene).

**Noncoding RNA**

RNA that does not code for protein but has some enzymatic, structural, or regulatory function. Although ribosomal RNA (rRNA) and transfer RNA (tRNA) are also untranslated RNAs, they are not included in this scope.

**Phenome/Phenomics**

The phenotypic counterpart or expression of the genome; the complete set of phenotypic characteristics of an organism.

**Precision breeding**

A genetic engineering technique of reproducing a species member to retain desirable traits and produce a stronger hybrid.

**Probiotics**

Live, nonpathogenic, nontoxic microbial organisms that confer a health benefit on the host when administered in adequate amounts.

**Proteomics**

Technologies that characterize the translated (protein) portion of the animal genome, including all subsequent covalent and structural modifications of those proteins.

**Quantitative genetics**

The area of genetics concerned with the inheritance of continuously varying traits. Most practical improvement programs involve the application of quantitative genetics.

**Regulatory elements (promoters, enhancers, repressors)**

In genetics, a promoter is a region of DNA that initiates transcription of a particular gene.

In genetics, an enhancer is a short (50- to 1500-base pair) region of DNA that can be bound by proteins (activators) to activate transcription of a gene.

In molecular genetics, a repressor is a DNA- or RNA-binding protein that inhibits the expression of one or more genes by binding to the operator or associated silencers. A DNA-binding repressor blocks the attachment of RNA polymerase to the promoter, thus preventing transcription of the genes into messenger RNA.

**Single nucleotide polymorphism**

A single nucleotide variation in a genetic sequence that occurs at appreciable frequency in the population.

**SNP assay**

The measurement of genetic variations of single nucleotide polymorphisms (SNPs) between members of a species.

**Stem cells**

Undifferentiated cells that retain the ability to self-regenerate and differentiate into specialized cells.

**Symbiosis/dysbiosis**

Changes in quantitative and qualitative composition of microbiota. The changes may lead to altered host microbial interaction or homeostatic imbalance that can contribute to a disease state, often with inflammation.

**Systems biology**

The use of quantitative measurements of the behavior of groups of interacting components; systematic measurement technologies such as genomics, bioinformatics, and proteomics; and mathematical and computational models to describe and predict dynamical behavior.

**Traits or phenotypes**

The visible and/or measurable characteristics of an organism (how it appears outwardly).

**Transcript**

A length of RNA or DNA that has been transcribed respectively from a DNA or RNA template.

**Transcriptome**

An organism’s complete set of mRNA molecules. The transcriptome may vary depending upon the condition and environment of the organism.

**Transcriptomics**

Technologies that characterize the transcribed (RNA) portion of the animal genome.

**Further genomics-related definitions can be found at these web sites:**

https://www.usda.gov/topics/biotechnology/biotechnology-glossary

https://agclass.nal.usda.gov/glossary_az.shtml

https://www.bio.org/articles/glossary-agricultural-biotechnology-terms

http://www.fao.org/docrep/003/x3910e/x3910e04.htm

## Appendix 1: Steering Committee

National Program Leaders at ARS and NIFA assembled a steering committee to aid in the development of the workshop and participate in leading the writing of the current Blueprint. The steering committee included representatives of land grant universities, ARS scientists, Office of National Programs and industry. Several members provided continuity across previous and current Blueprint activities as they had also served on the steering committee for the previous document.

**USDA National Program Leadership**

- Dr. Jeffrey Vallet, ARS, Beltsville, Maryland
- Dr. Lakshmi Kumar Matukumalli, NIFA, Washington, DC
- Dr. Caird Rexroad, ARS, Beltsville, Maryland

**USDA-ARS Scientists**

- Dr. Hans Cheng, Avian Disease and Oncology Laboratory, East Lansing, Michigan
- Dr. Joan Lunney, Animal Parasitic Diseases Laboratory, Beltsville, Maryland
- Dr. Tim Smith, U.S. Meat Animal Research Center, Clay Center, Nebraska
- Dr. Curt Van Tassell, Animal Genome Improvement Laboratory, Beltsville, Maryland

**University Faculty**

- Dr. Noelle Cockett, Utah State University, Logan, Utah
- Dr. John Liu, Syracuse University, Syracuse, New York
- Dr. James Reecy, Iowa State University, Ames, Iowa
- Dr. Jerry Taylor, University of Missouri, Columbia, Missouri
- Dr. Catherine Ernst, Michigan State University, East Lansing, Michigan

**Industry Representative**

- Dr. Janet Fulton, Hy-Line International, West Des Moines, Iowa

**USDA Program Support**

- Ms. Janice Boarman, ARS, Beltsville, Maryland
- Ms. Loren Coleman, ARS, Beltsville, Maryland

## Appendix 2: Writing Teams

The Steering Committee identified additional members of the animal genomics community who could participate in drafting the current Blueprint. The Steering Committee identified important topics and developed the first draft. At the workshop, writing teams continued to develop the draft which the ARS national program leaders continued to refine. The document was presented in an online platform in May 2018 to solicit broader input, including international review.

**Science to Practice**

***Optimizing Animal Production through Precision Breeding and Management***

**Team Leaders:** Dr. Jerry Taylor, Dr. Curt Van Tassell, Dr. Mark Boggess,

Dr. Archie Clutter

**Team Members** Dr. Larry Kuehn, Dr. Gary Rohrer, Dr. Juan Steibel, Dr. Guilherme Rosa, Dr. Elaine Grings

**Discovery Science**

***Understanding Genome Biology to Accelerate Genetic Improvement of Economically Important Traits***

**Team Leaders:** Dr. Joan Lunney, Dr. Cathy Ernst

**Team Members:** Dr. Samantha Brooks, Dr. Brenda Murdoch, Dr. Daniel Ciobanu,

Dr. Chris Tuggle, Dr. Huaijun Zhou, Dr. Chris Seabury

***Reducing the Impacts of Livestock Diseases***

**Team Leaders:** Dr. Hans Cheng, Dr. Holly Neibergs

**Team Members:** Dr. Tim Kurt, Dr. Sue Lamont, Dr. Stephen White, Dr. Millie Worku,

Dr. Graham Plastow

***Applying Precision Agriculture Technologies to Livestock Phenotyping***

**Team Leaders:** Dr. Jim Reecy, Dr. Joan Lunney

**Team Members:** Dr. Erin Connor, Dr. Molly McCue, Dr. Jennifer Woodward-Greene,

Dr. Nick Serao

***Harnessing the Microbiome to Improve the Efficiency and Sustainability of Livestock Production***

**Team Leaders:** Dr. Derek Bickhart, Dr. Tim Smith

**Team Members:** Dr. Deb Hamernik, Dr. Samodha Fernando, Dr. Stephen Schmitz-Esser

**Infrastructure**

***Developing Advanced Genomic Tools, Technologies and Resources for Agricultural Animals***

**Team Leaders:** Dr. Tim Smith, Dr. Hans Cheng, Dr. John Liu

**Team Members:** Dr. Stephanie McKay, Dr. Clare Gill, Dr. Yniv Palti, Dr. Robert Schnabel,

Dr. Catherine Purcell

***Training the Next Generation of Animal Scientists***

**Team Leaders:** Dr. Cathy Ernst, Dr. John Liu

**Team Members:** Dr. Penny Riggs, Dr. Mohamed Salem, Dr. Brian Sayre, Dr. David Bailey

***Advancing Biotechnology to Improve the Sustainability and Efficiency of Animal Production***

**Team Leaders:** Dr. Curt Van Tassell, Dr. Jim Reecy

**Team Members:** Dr. Christine Elsik, Dr. Brittany Keel, Dr. James Koltes,

Dr. Jennifer Weller

***Advancing Biotechnology to Improve the Sustainability and Efficiency of Animal Production***

**Team Leaders:** Dr. Tad Sonstegard, Dr. Noelle Cockett, Dr. Kevin Wells,

Dr. Bhanu Telugu

**Team Members:** Dr. Kiho Lee, Dr. Chris Hostetler, Dr. Carl Schmidt,

Dr. Alison Van Eenennaam

***Characterizing and Preserving Genetic Diversity for the Future of Animal Production***

**Team Leaders:** Dr. Harvey Blackburn, Dr. Noelle Cockett, Dr. Janet Fulton

**Team Members:** Dr. Jared Decker, Dr. Julie Long, Dr. Phil Sponenberg, Dr. Alison Martin

## Appendix 3: Workshop

The workshop entitled “Genome to Phenome: A USDA Blueprint for Improving Animal Production” was held November 15-16, 2017 at the National Agricultural Library in Beltsville, Maryland. This workshop was an effort to update the previous report, “Blueprint for USDA Efforts in Agricultural Animal Genomics 2008–2017”*,* which was developed by the animal genomics community under the leadership of Dr. Ronnie Green (USDA-ARS) and Dr. Muquarrab Qureshi (USDA-NIFA). Over the last decade the vision outlined in this document served to guide intramural and extramural research programs at USDA and in the broader international community. This workshop revisited progress to date and developed meaningful and tangible goals for the next decade by developing the second-generation blueprint for the animal genomics community. The workshop program is presented below, and attendees are listed in Appendix 4.

Workshop participants were selected to represent diversity with respect to their institutions, geography, stage of career, scientific discipline and species of interest. Participants represented:

- Land Grant Universities
- Minority Serving Institutions
- Federal Science Agencies
- Animal Agriculture Industries
- International Colleagues
- Non-Governmental Organizations

Presentations included comments from ARS and NIFA leaders, industry stakeholders, and leading scientists from outside the agriculture sector in their respective fields of genome biology.

The program, including links to video presentations, can be found at:

<https://www.animalgenome.org/share/meetings/Genome2Phenome>

**
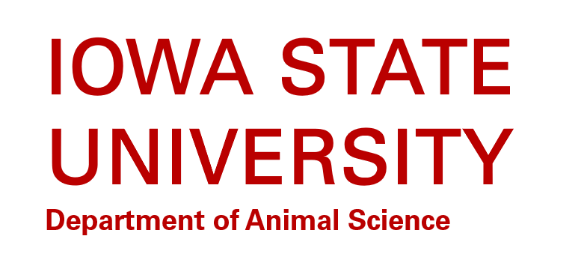

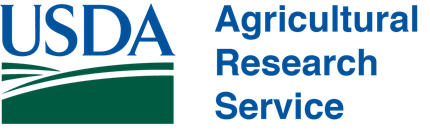
**

**Genome to Phenome: A USDA Blueprint for Improving Animal Production**

**National Agricultural Library, Beltsville, Maryland**

**November 15 and 16, 2017**

**AGENDA**

**This workshop is supported by AFRI grant 2017-67015-26907 project accession** [**1013525**](https://portal.nifa.usda.gov/web/crisprojectpages/1013525.php) **from the USDA National Institute of Food and Agriculture*,* the ARS Office of National Programs, and the National Agricultural Library.**

**Wednesday November 15, 2017**

**8:30 am** Arrive, process thru security

**9:00 am Welcome**

Dr. Caird Rexroad (USDA-ARS), Dr. Jeff Vallet (USDA-ARS) and

Dr. Adele Turzillo (USDA-NIFA), Dr. Lakshmi Matukumalli (USDA-NIFA)

**9:05 am Logistics**

Dr. Caird Rexroad, USDA ARS

**9:10 am** [The 2008 Blueprint for USDA Efforts in Agricultural Animal Genomics](https://www.animalgenome.org/share/meetings/Genome2Phenome.video?f=Ronnie%20Green)

Dr. Ronnie Green, Chancellor, University of Nebraska

**9:20 am** **Implementing Blueprint at the USDA**

[Dr. Jeffrey Silverstein, USDA ARS](https://www.animalgenome.org/share/meetings/Genome2Phenome.video?f=Jeffrey%20Silverstein)

[Dr. Parag Chitnis, USDA NIFA](https://www.animalgenome.org/share/meetings/Genome2Phenome.video?f=Parag%20Chitnis)

**9:40 am Implementing the Blueprint with Federal and International Partners (Introductions)**

Dr. Jennifer Weller (DBI) NSF

Dr. Neelakanta Ravindranath (NICHD) and Dr. Dan Gilchrist (NHGRI), NIH

Dr. Ramana Madupu, DOE

Dr. Lindsay Parish USAID

Dr. Tim Kurt, FFAR

Dr. Jean-Charles Cavitte, European Commission

Dr. David Bailey, Genome Canada

**9:50 am** [Developing a Next Generation Blueprint](https://www.animalgenome.org/share/meetings/Genome2Phenome.video?f=Caird%20Rexroad)

Dr. Caird Rexroad, USDA ARS

**10:00 am**  [Livestock High-Throughput Phenotyping and Big Data Analytics](https://www.animalgenome.org/share/meetings/Genome2Phenome.video?f=James%20Reecy)

Dr. James Reecy, Iowa State University

| **10:40 am Livestock Industry Perspectives**   \| - [American Horse Council](https://www.animalgenome.org/share/meetings/Genome2Phenome.doc/01.AmericanHorse.pdf) - [American Paint Horse Association](https://www.animalgenome.org/share/meetings/Genome2Phenome.doc/02.AmericanPaintHorse.pdf) - [American Quarter Horse Association](https://www.animalgenome.org/share/meetings/Genome2Phenome.doc/03.AmericanQuarterHorse.pdf) - [Angus Genetics, Inc](https://www.animalgenome.org/share/meetings/Genome2Phenome.video?f=Dan%20Moser) - [Arabian Horse Association](https://www.animalgenome.org/share/meetings/Genome2Phenome.doc/05.ArabianHorseAssoc.pdf) - [Aviagen Group](https://www.animalgenome.org/share/meetings/Genome2Phenome.doc/06.Aviagen.pdf) - [Genesus](https://www.animalgenome.org/share/meetings/Genome2Phenome.doc/07.Genesus.pdf) - [Hendrix Genetics](https://www.animalgenome.org/share/meetings/Genome2Phenome.doc/08.HendrixGenetics.pdf) \| - [Joe Jurgielewicz and Son LTD](https://www.animalgenome.org/share/meetings/Genome2Phenome.doc/09.JoeJurgielewicz.pdf) - [Select Sires](https://www.animalgenome.org/share/meetings/Genome2Phenome.doc/10.SelectSires.pdf) - [S’Klallam Tribe, Sequim, WA](https://www.animalgenome.org/share/meetings/Genome2Phenome.doc/11.KlallamTribe.pdf) - [Superior Farms](https://www.animalgenome.org/share/meetings/Genome2Phenome.doc/12.Superior.pdf) - [The Jockey Club](https://www.animalgenome.org/share/meetings/Genome2Phenome.doc/13.JockeyClubEquine.pdf) - [Transova](https://www.animalgenome.org/share/meetings/Genome2Phenome.doc/14.Transova.pdf) - [USTrotting Association](https://www.animalgenome.org/share/meetings/Genome2Phenome.doc/15.TrottingAsso.pdf) - [Welsh Pony and Cob Society of America](https://www.animalgenome.org/share/meetings/Genome2Phenome.doc/16.WelshPony.pdf) \| \| --- \| --- \| |
| --- | --- | --- |

**11:30 am Discussion Session I: *Science to Practice***

| **Precision Selection 1** | **Precision**  **Selection 2** | **Precision Management Systems 1** | **Precision Management Systems 2** |
| --- | --- | --- | --- |
| Dr. Curt Van Tassell  Dr. Caird Rexroad  *Sara Nilson*  *Luke Kramer* | Dr. Jerry Taylor  Dr. Lakshmi Kumar Matukumalli  *Troy Rowan* | Dr. Mark Boggess  Dr. Jeffrey Silverstein  *Kaitlyn Daza* | Dr. Archie Clutter  Dr. Jeffrey Vallet  *Bethany Krehbiel* |

**12:00 pm Working Lunch**

**1:00 pm** [Thoughts on Components of an Agricultural Data Ecosystem](https://www.animalgenome.org/share/meetings/Genome2Phenome.video?f=Sean%20Davis)

Dr. Sean Davis, NIH

**1:40 pm** [Long Reads and the Future of Vertebrate Genome Sequencing](https://www.animalgenome.org/share/meetings/Genome2Phenome.video?f=Adam%20Phillippy)

Dr. Adam Phillippy, NIH

**2:20 pm Discussion Session II: *Discovery Science***

| **Genomic and Functional Biology** | **Host Pathogen Interactions** | **Phenotyping** | **Microbiome and Metagenomics** |
| --- | --- | --- | --- |
| Dr. Cathy Ernst  Dr. Joan Lunney  *Troy Rowan* | Dr. Hans Cheng  Dr. Holly Neibergs  *Sara Nilson* | Dr. Jeffrey Vallet  Dr. Jim Reecy  *Bethany Krehbiel*  *Kaitlyn Daza* | Dr. Derek Bickhart  Dr. Tim Smith  *Luke Kramer* |

**3:50 pm Report Out – Discussion Sessions I and II**

**4:30 pm Panel Discussion – Federal and International Partners**

**5:00 pm Adjourn**

**Thursday November 16, 2017**

**8:30 am** Arrive, process thru security

**9:00 am** [Writing Genomes](https://www.animalgenome.org/share/meetings/Genome2Phenome.video?f=Jef%20Boeke)

Dr. Jef Boeke, New York Medical Center

**9:40 am** [Using Networks to Understand the Genotype-Phenotype Connection](https://www.animalgenome.org/share/meetings/Genome2Phenome.video?f=John%20Quackenbush)

Dr. John Quackenbush, Harvard T.H. Chan School of Public Health

**10:20 am Discussion Session III: *Infrastructure***

| **Genomic Tools and Resources** | **Education and Training** | **Bioinformatics and Computational Biology** | **Genome Modification** | **Animal Populations/ Germplasm Preservation** |
| --- | --- | --- | --- | --- |
| Dr. Tim Smith  Dr. Hans Cheng  *Troy Rowan* | Dr. Cathy Ernst  Dr. John Liu  *Sara Nilson* | Dr. Jim Reecy  Dr. Curt Van Tassell  *Luke Kramer* | Dr. Kevin Wells  Dr. Tad Sonstegard  Dr. Bhanu Telugu  *Kaitlyn Daza* | Dr. Janet Fulton  Dr. Harvey Blackburn  Dr. Noelle Cockett  *Bethany Krehbiel* |

**11:30 am Report Out Discussion Session III**

**12:00 pm Working Lunch**

[Presentation from the National Agricultural Library](https://www.animalgenome.org/share/meetings/Genome2Phenome.video?f=Cynthia%20Parr)

Dr. Cynthia Parr

**1:00 pm Writing Teams I – See Pre-Workshop Draft**

| **Genomic and Functional Biology** | **Host-Pathogen Interactions** | **Microbiome** | **Genome Modification** | **Bioinformatics and Computational Biology** |
| --- | --- | --- | --- | --- |

**2:30 pm Writing Teams II – See Pre-Workshop Draft**

| **Phenotyping** | **Genomic Tools and Resources** | **Education and Training** |
| --- | --- | --- |
| **Precision**  **Selection** | **Precision Management Systems** | **Animal Populations/Germplasm Preservation** |

**4:00 pm** [Discussion Sessions Report Out](https://www.animalgenome.org/share/meetings/Genome2Phenome.video?f=Discussion%20Report)

[Writing Teams Report Out](https://www.animalgenome.org/share/meetings/Genome2Phenome.video?f=Writing%20Report)

**5:00 pm Conclude**

## Appendix 4: Workshop Participants

| **Participant Name** | **Affiliation** |
| --- | --- |
| Bailey, David | Genome Alberta/Canada |
| Bickhart, Derek | USDA, ARS, DFRC - Madison, WI |
| Blackburn, Harvey | USDA, ARS, NAGP - Fort Collins, CO |
| Boeke, Jef | NYU |
| Brooks, Samantha | University of Florida |
| Cavitte, Jean-Charles | European Commission DG Agriculture and Rural Development |
| Childers, Anna | USDA, ARS, BRL - Beltsville, MD |
| Childers, Chris | USDA, ARS, NAL - Beltsville, MD |
| Chitnis, Parag | USDA, NIFA |
| Cockett, Noelle | Utah State University |
| Connor, Erin | USDA, ARS, BARC - Beltsville, MD |
| Davis, Sean | NIH |
| Daza, Kaitlyn | Michigan State University |
| Decker, Jared | University of Missouri |
| Elsik ,Christine | University of Missouri |
| Ernst, Cathy | Michigan State University |
| Fernando, Samodha | University of Nebraska |
| Fulton, Janet | Hyline |
| Gilchrist, Dan | NIH, NHGRI |
| Gill, Clare | Texas A&M University |
| Grings, Elaine | USAID |
| Hamernik, Deb | University of Nebraska |
| Hostetler, Chris | National Pork Board |
| Hu, Zhiliang | Iowa State University |
| Jennifer, Weller | NSF-BIO/DBI |
| Keel, Brittany | USDA, ARS, USMARC - Clay Center, NE |
| Koltes, James | Iowa State University |
| Kramer, Luke | Iowa State University |
| Krehbiel, Bethany | Colorado State University |
| Kuehn, Larry | USDA, ARS, USMARC - Clay Center, NE |
| Kurt, Tim | FFAR |
| Lamont, Susan | Iowa State University |
| Lee, Kiho | Virginia Tech |
| Liu, John | Syracuse University |
| Long, Julie | USDA, ARS BARC - Beltsville, MD |
| Lunney, Joan | USDA, ARS, BARC - Beltsville, MD |
| Martin, Alison | Livestock Conservancy |
| Martinez, Gonzalo | Choice-Genetics |
| Matukumalli, Lakshmi Kumar | USDA, NIFA - Washington, DC |
| McCue, Molly | University of Minnesota |
| McKay, Stephanie | University of Vermont |
| Moore, Eli | USDA, ARS, NAL - Beltsville, MD |
| Murdoch, Brenda | University of Idaho |
| Neibergs, Holly | Washington State University |
| Nilson, Sara | University of Missouri |
| Palti, Yniv | USDA, ARS, NCCCWA - Kearneysville, WV |
| Parish, Lindsay | USAID |
| Phillippy, Adam | NIH |
| Plastow, Graham | University of Alberta |
| Quackenbush, John | Harvard T.H. CHAN School of Public Health |
| Ravindranath, Neelakanta | NICHD |
| Reecy, James | Iowa State University |
| Rexroad, Caird | USDA, ARS, ONP, APP - Beltsville, MD |
| Riggs, Penny | Texas A&M University |
| Rohrer, Gary | USDA, ARS, USMARC - Clay Center, NE |
| Rosa, Guilherme | University of Wisconsin |
| Rowan, Troy | University of Missouri |
| Salem, Mohamed | Middle Tennessee State University |
| Sayre, Brian | Virginia State University |
| Schmidt, Carl | University of Delaware |
| Schnabel, Robert | University of Missouri |
| Seabury, Christopher | Texas A&M University |
| Serão, Nick | Iowa State University |
| Silverstein, Jeffrey | USDA, ARS, ONP, APP - Beltsville, MD |
| Smith, Tim | USDA, ARS, USMARC - Clay Center, NE |
| Sonstegaard, Tad | Acceligen |
| Steibel, Juan | Michigan State University |
| Taylor, Jerry | University of Missouri |
| Telugu, Bhanu | University of Maryland |
| Tuggle, Chris | Iowa State University |
| Turzillo, Adele | USDA, NIFA - Washington, DC |
| Vallet, Jeff | USDA, ARS, APP - Beltsville, MD |
| Van Tassell, Curt | USDA, ARS, BARC - Beltsville, MD |
| Wells, Kevin | University of Missouri |
| White, Stephen | USDA, ARS, ADRU - Pullman, WA |
| Woodward-Greene, Jennifer | USDA, ARS, OIRP - Beltsville, MD |
| Worku, Mulumebet | North Carolina A&T State University |
| Zhou, Huaijun | University of California - Davis |

## Appendix 5 Animal Genome Information

A search of PubMed for publications on the 13 species highlighted in the previous Blueprint whose titles includes terms associated with genomics using the following search criteria:

(*genom* OR sequenc* OR GWAS OR SNP OR polymorphi* OR transcript* OR *DNA* OR *RNA*) AND (species OR common name).


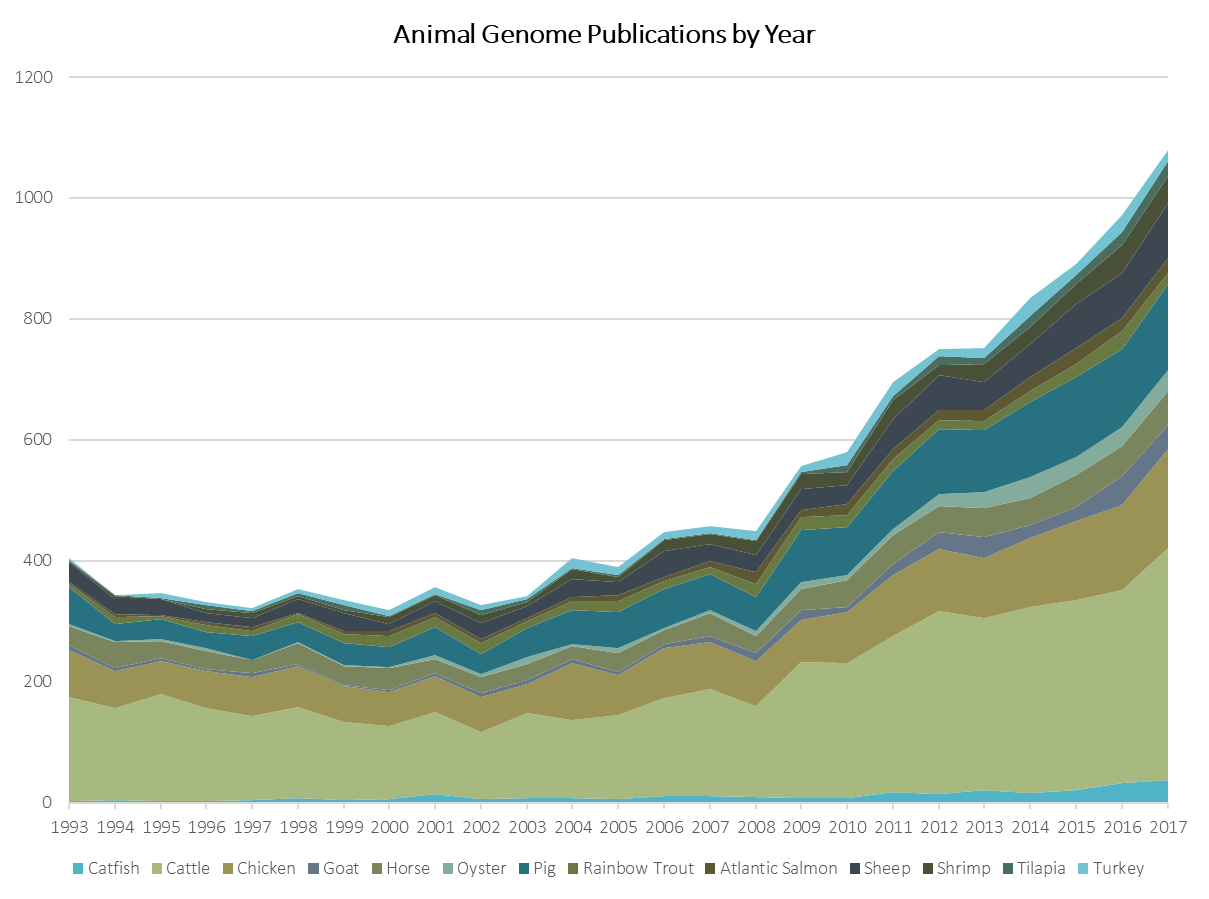


5a. Increases in numbers of genomics publications for 13 species highlighted in the 2008 Blueprint.


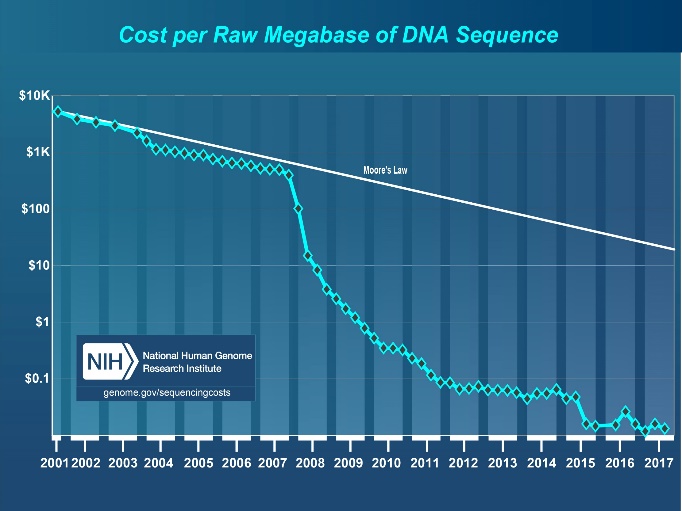


5b. Wetterstrand KA. DNA Sequencing Costs: Data from the NHGRI Genome Sequencing Program (GSP) Available at: [www.genome.gov/sequencingcostsdata](https://www.genome.gov/sequencingcostsdata).

5c. Increase in the volume of single nucleotide polymorphism data characterizing genetic diversity and creating tools for genomic selection and understanding the biology of traits.

5d. Increases in submissions to the NIH National Library of Medicines Short Read Archive for 13 animal species highlighted in the 2008 Blueprint.

**QTLdb**


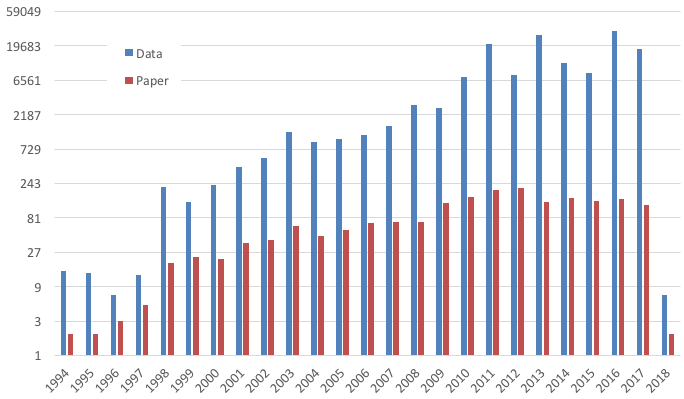


5e. Increase in submissions of QTL data and journal articles describing associations of genome data with traits https://www.animalgenome.org/cgi-bin/QTLdb/index.

## Appendix 6 Animal Germplasm Preservation

The inventory of germplasm preserved by the USDA ARS National Animal Germplasm Program <https://nrrc.ars.usda.gov/A-GRIN/main_webpage_dev/ars?record_source=US> has increased dramatically since in inception.


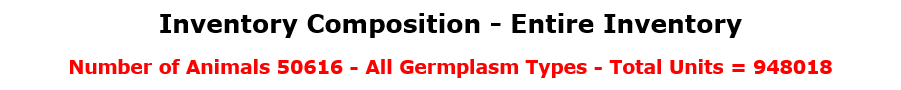


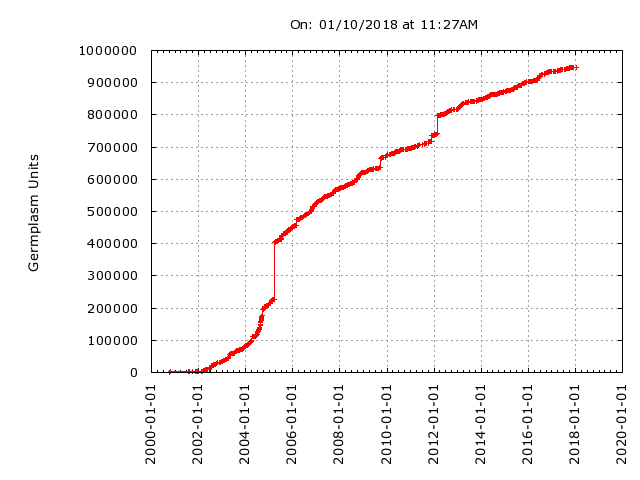

Supplement: Supplementary file 1 [file Table_1.DOCX]
